# Biohythane production via anaerobic digestion process: fundamentals, scale-up challenges, and techno-economic and environmental aspects

**DOI:** 10.1007/s11356-024-34471-8

**Published:** 2024-08-01

**Authors:** Seyedeh Azadeh Alavi-Borazjani, Luís António da Cruz Tarelho, Maria Isabel Capela

**Affiliations:** Department of Environment and Planning/Centre for Environmental and Marine Studies (CESAM), University of Aveiro, Campus Universitário de Santiago, 3810-193 Aveiro, Portugal

**Keywords:** Biohydrogen, Biomethane, Renewable energy, Biorefinery, Waste management, Circular economy

## Abstract

Biohythane, a balanced mixture comprising bioH_2_ (biohydrogen) and bioCH_4_ (biomethane) produced through anaerobic digestion, is gaining recognition as a promising energy source for the future. This article provides a comprehensive overview of biohythane production, covering production mechanisms, microbial diversity, and process parameters. It also explores different feedstock options, bioreactor designs, and scalability challenges, along with techno-economic and environmental assessments. Additionally, the article discusses the integration of biohythane into waste management systems and examines future prospects for enhancing production efficiency and applicability. This review serves as a valuable resource for researchers, engineers, and policymakers interested in advancing biohythane production as a sustainable and renewable energy solution.

## Introduction

The rapid increase in energy consumption is depleting natural resources and causing a significant rise in greenhouse gas emissions. To tackle the challenges posed by fossil fuels, the world is shifting towards a more sustainable and cleaner energy system. Recent statistics show that global renewable energy capacity reached 3,869,705 megawatts (MW) in 2023, marking a 128% increase from 2014 (IRENA 2024). This growth is attributed to various renewable energy sources, as detailed in Table 1. Furthermore, global renewable energy consumption has surged, with levels nearing 45.18 exajoules in 2022, a notable 227% increase from 2012 (Energy Institute 2023).

**Table 1:** Global renewable energy capacity in 2023 and percentage increase from 2014

Renewable energy source	Capacity in 2023 (MW)	Percentage increase from 2014
Hydropower	1,407,754	19.6%
Marine energy	527	2.5%
Wind energy	1,017,199	191.1%
Solar energy	1,418,969	685.0%
Bioenergy	150,261	65.4%
Geothermal energy	14,846	32.0%

Source: IRENA (2024)

To develop a more sustainable energy system, the use of hydrogen (H_2_) is being explored extensively. H_2_ is considered a highly efficient and clean energy carrier with a calorific value of 142 MJ/kg and only water as a combustion by-product (Silva et al. 2017). Methane (CH_4_) is also being investigated as a clean and safe alternative to petroleum and diesel, widely utilized in various industries such as chemical and transport sectors (Abdur Rawoof et al. 2021). Unlike traditional fuels, the combustion of H_2_ and CH_4_ does not result in the emission of major air pollutants like nitrous oxides (NO_X_) and sulfur dioxide (SO_2_) (Krishnan et al. 2019; Mozhiarasi et al. 2023). However, CH_4_ has limitations such as slow burning speed, narrow flammability range, and high ignition temperature, leading to lower combustion efficiency and higher energy consumption in CNG-powered vehicles (Bauer and Forest 2001). By adding even a small amount of H_2_ to CH_4_, the flammability range is extended, and ignition time is reduced due to H2’s higher mass-specific heating value and faster flame speed compared to CH_4_. Additionally, mixing H_2_ with CH_4_ increases the hydrogen-to-carbon ratio, resulting in decreased greenhouse gas emissions (Hans and Kumar 2019). Furthermore, blending CH_4_ with H_2_ reduces the risks of abnormal combustion like flashback and engine explosion, common issues in H_2_ combustion (Makaryan et al. 2022).

Hythane, a blend of H_2_ and CH_4_, has emerged as a promising energy carrier for the transition to a hydrogen-based economy. This valuable energy carrier was initially developed by Hydrogen Component Inc. (HCI) in the early 1990s as a fuel for powering internal combustion engines (Bolzonella et al. 2018). In 2004, the patent and trademark for hythane were acquired by Eden Energy, leading to the establishment of Hythane™ Company LLC (now Eden Innovations LLC). Following this acquisition, several pilot-scale projects were launched globally (McWilliams 2017). The Montreal hythane bus project successfully reduced NO_x_ emissions by utilizing hythane with a 10% (v/v) H_2_ content. Compared to CNG-powered buses, the use of hythane resulted in a 45% decrease in NO_x_ emissions. Similarly, the Sun Line transit agency project in California demonstrated the environmental benefits of hythane as an alternative fuel. By using hythane with over 20% (v/v) H_2_ content, the project achieved a significant 50% reduction in NO_x_ emissions compared to CNG-powered buses (Abdur Rawoof et al. 2021). The Fiat Company also developed a car with a 900 cc two-cylinder engine using hythane with 30% H_2_ (v/v), achieving low CO_2_ emissions of only 69 g/km^2^ (Bolzonella et al. 2018). Some of the proven advantages of hythane in the automotive sector include lower energy requirements for engine combustion and increased heat and fuel efficiency (Abdur Rawoof et al. 2021). Additionally, the use of hythane as a vehicle fuel does not necessitate any special storage system or significant modifications to existing CNG engines and infrastructure (Krishnan et al. 2019). The superior properties of hythane have led to its commercialization as a transport fuel in various countries, including the USA and India. Furthermore, its potential as an alternative fuel source has attracted the interest of major automobile manufacturers, such as Fiat, Volvo, and Toyota (Shanmugam et al. 2021a). Table 2 presents a summary of the advantages and disadvantages associated with hythane.

**Table 2:** Main advantages and disadvantages of hythane

Advantages	Disadvantages	Ref.
- Increased flame speed and reduced combustion duration - Overcoming high ignition temperatures - Higher thermal efficiency compared to natural gas - Greater braking efficiency and power output over natural gas - Minimization of cycle-by-cycle variations in engines - Reduced fuel consumption - Reducing emissions of unburned hydrocarbons, CO_2_, and CO - Possible utilization without significant modifications to existing natural gas infrastructure and CNG engines	- Potential for increased NO_x_ emissions at higher hydrogen contents without adequate tuning - Dependence of total CO_2_ emissions on the method of hydrogen production - Increased heat transfer to cylinder walls and a decrease in engine power - Increased ignition delay time at higher hydrogen contents, necessitating careful engine calibration - Need for careful handling and storage due to hydrogen’s flammability and diffusivity - Requirement for modifying existing storage and supply infrastructure to handle higher hydrogen contents	Akansu et al. (2004); Genovese et al. (2011); Villante and Genovese (2012); Biffiger and Soltic (2015); Yadav and Sircar (2017); Makaryan et al. (2022)

**Fig. 1:**
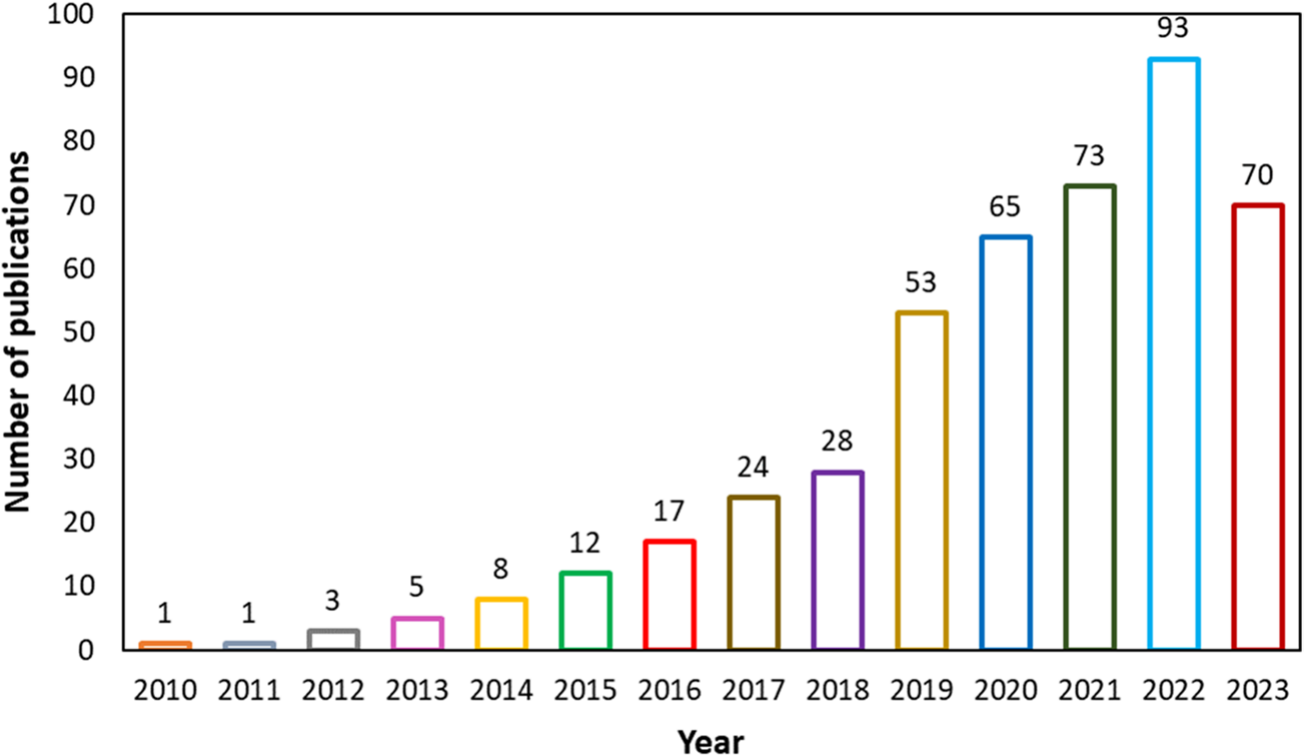
Publication trends for “biohythane” on Science Direct (2010–2023)

Currently, hythane production is mainly done using energy-intensive thermochemical processes that rely on fossil fuels like natural gas (Shanmugam et al. 2021a). However, there is a shift towards more sustainable methods, including biological production, to address the drawbacks of these processes. The biological production of hythane from renewable resources like biomass through anaerobic digestion (AD) offers significant sustainability advantages over fossil fuel-based processes (Alavi-Borazjani et al. 2023). This process can produce both H_2_ and CH_4_, which can be used independently or combined to create biohythane, a biologically derived hythane mixture (Liu et al. 2013). The potential benefits of biological hythane production have spurred active research in the field. As shown in Fig. 1, from 2010 to 2022, there was a consistently increasing trend in biohythane publications, rising from only one in 2010 to 92 in 2022. However, in 2023, there was a slight decrease, with 70 articles published according to data from the Science Direct database. Despite this slight dip, the overall trend indicates a growing interest in biohythane research, emphasizing the need for continued investment and exploration in this area.

This comprehensive review pushes the boundaries of biohythane production research by moving beyond the existing body of knowledge. While acknowledging the important groundwork laid by previous reviews, it dives deeper into the topic, carefully selecting distinct and crucial information from a wider array of recent studies. By presenting data and insights not previously covered, this work offers a fresh perspective on the multifaceted domain of biohythane production via the AD process. This meticulous approach aims to provide researchers with a thorough and up-to-date one-stop resource, streamlining the process of literature searches and cross-referencing. As a result, this review serves as a valuable tool, equipping researchers with a well-rounded perspective on the current landscape of biohythane generation through AD.

## Mechanisms of biohythane production

**Fig. 2:**
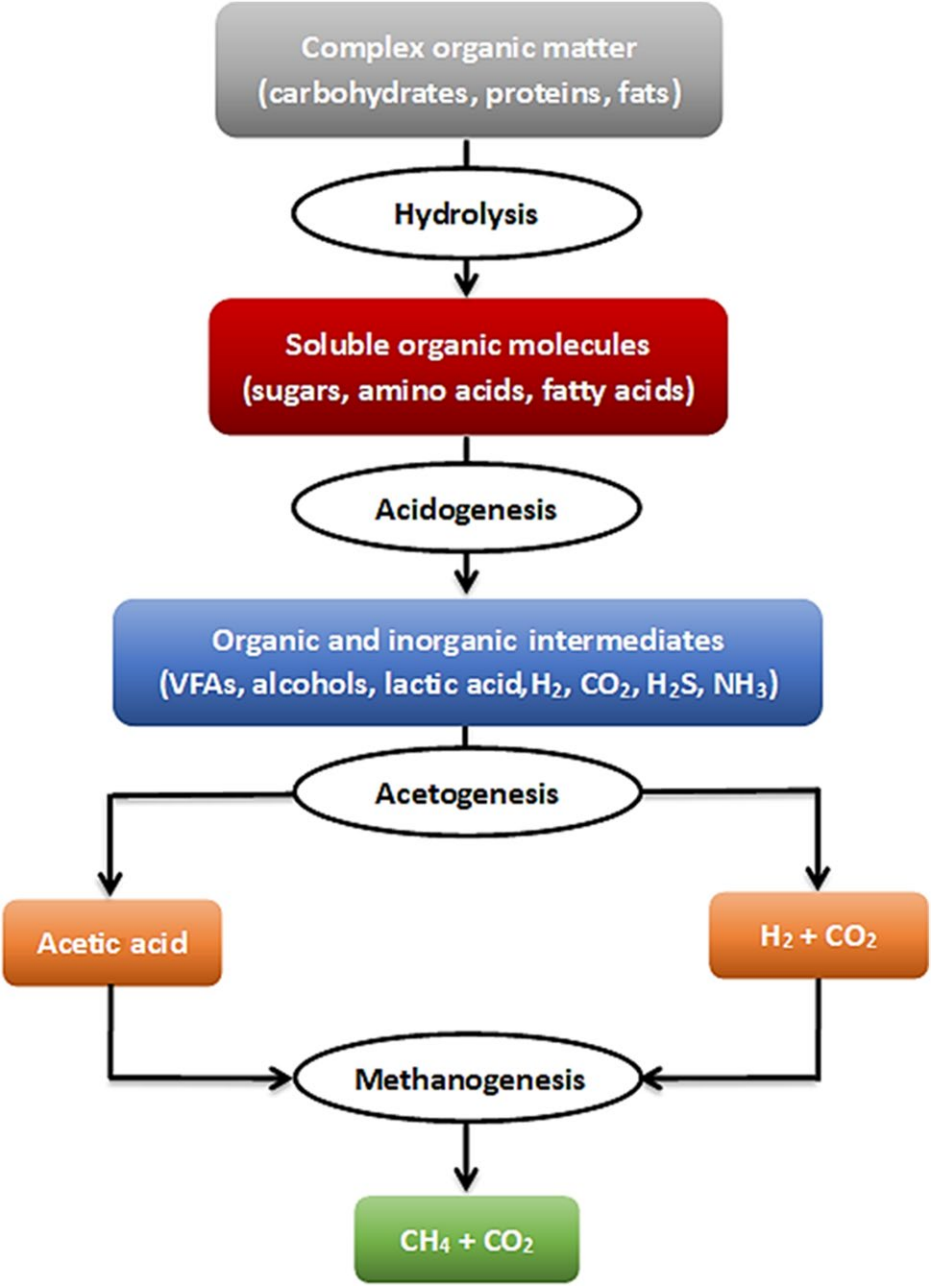
Main steps in the anaerobic digestion process

Anaerobic digestion (AD) involves the biological breakdown of organic matter by microorganisms in an oxygen-free environment, resulting in the production of biogas. The process typically consists of hydrolysis followed by acidogenesis, acetogenesis, and finally methanogenesis (Tsigkou et al. 2022). Figure 2 illustrates the key steps of the AD process.

During hydrolysis, complex organic compounds are enzymatically broken down into simpler constituents, including monosaccharides, amino acids, and fatty acids (Alavi-Borazjani et al. 2021a, 2021b). In the acidogenesis phase, the products of hydrolysis are transformed by acidogenic bacteria into a mixture of organic intermediates like volatile fatty acids (VFAs), lactic acid, and alcohols, as well as inorganic intermediates such as H_2_, CO_2_, hydrogen sulfide (H_2_S), and ammonia (NH_3_) (Madsen et al. 2011). In the acetogenesis phase, acetogenic bacteria further process the products from the previous step, leading to the production of acetic acid (CH_3_COOH), H_2_, and CO_2_. In the concluding phase, referred to as methanogenesis, diverse microorganisms consume acetate, CO_2_, and H_2_ to generate CH_4_ (Jain et al. 2015).

**Fig. 3:**
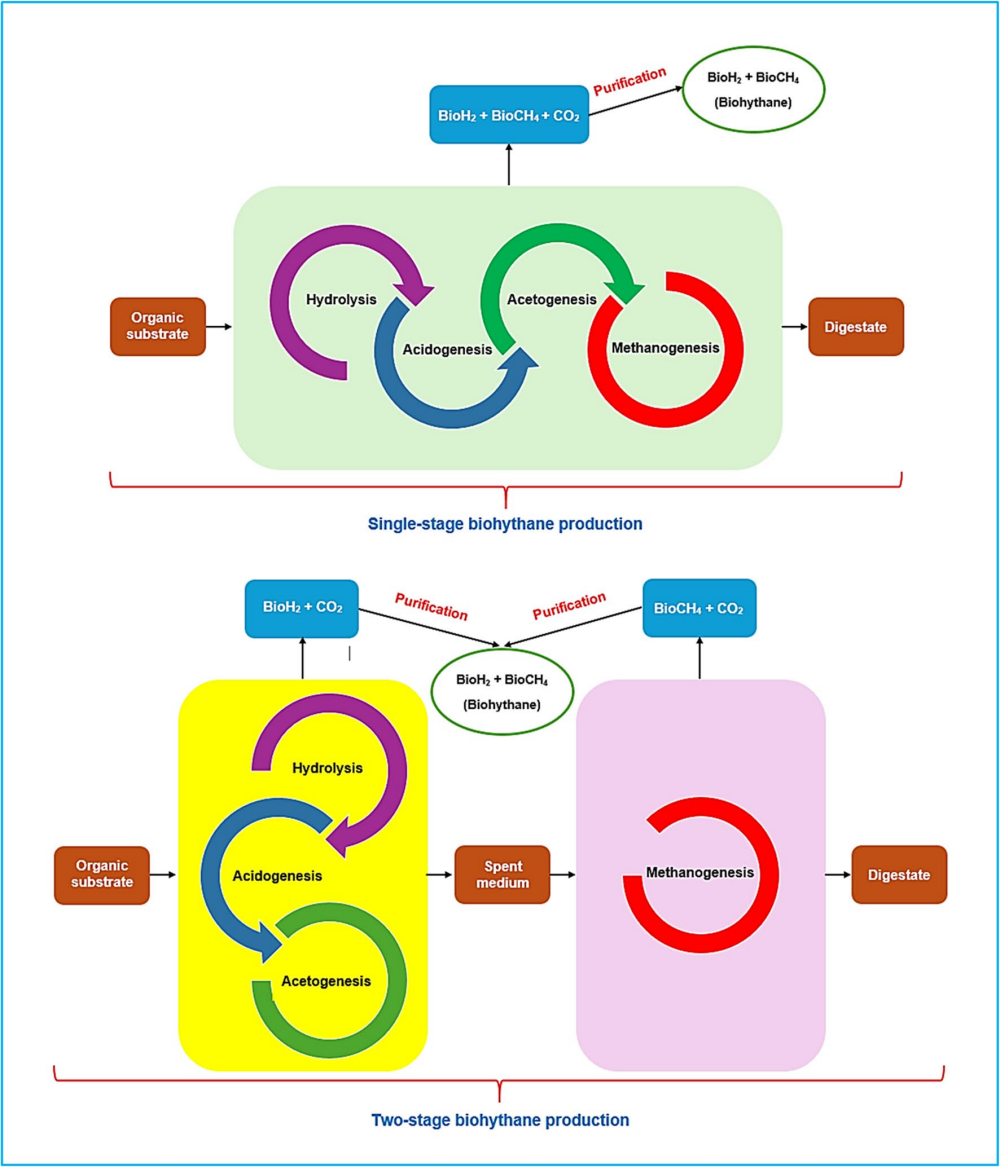
Single-stage and two-stage biohythane production

The synthesis of biohythane using the AD process can be achieved through either a single-stage or a two-stage approach, as shown in Fig. 3.

### Single-stage AD

In the single-stage AD process, the four stages of organic material conversion are integrated into a unified system (Nagao et al. 2012). The idea of co-producing bioH_2_ and bioCH_4_ in a single reactor to create sustainable hythane was proposed in 2015 (Ta et al. 2020). Single-stage AD offers advantages such as simplicity in design and reduced operating costs (Kabir et al. 2022). Research indicates that single-stage AD has led to interesting dynamics in microbial functions and interactions for biohythane production (Lay et al. 2020). However, a significant drawback is the risk of reactor acidification from excessive VFA formation during acidogenesis and acetogenesis. This can occur when VFA concentrations surpass a certain threshold, causing pH reduction, toxicity to hydrogen producers, and decreased H_2_ yield (Hans and Kumar 2019). To overcome the limitations of single-stage systems in biohythane production, a new two-compartment bioreactor has been developed (Vo et al. 2019). The upper and lower compartments serve specific roles in the production process, with the upper compartment dedicated to bioCH_4_ generation and the lower compartment focused on bioH_2_ refinement. This improved design allows for better control, enhanced reaction kinetics, and ultimately, superior biohythane production by segregating the two stages in separate compartments. Another promising approach in single-stage biohythane production involves entrapping hydrogenic and methanogenic bacteria separately (Ta et al. 2020; Nguyen et al. 2022). This concept entails trapping concentrated populations of hydrogenic and methanogenic bacteria within their respective beads. The use of the cell entrapment technique provides a protective environment for microbes while allowing substrate and metabolic product diffusion (Park and Chang 2000). By employing this method, the challenges associated with hydrogenotrophic methanogenesis can be mitigated, as the physical separation of hydrogenic and methanogenic bacteria enables better control and optimization of their metabolic activities (Ta et al. 2020).

### Two-stage AD

The two-stage AD concept was originally introduced by Travis in 1904 for wastewater treatment and has since been adapted by industries for both pre-treatment and post-treatment purposes (Rajendran et al. 2020). Recently, there has been a growing interest in applying this approach to biohythane production. In this process, the initial stages—hydrolysis, acidogenesis, and acetogenesis—are combined in a single reactor to produce bioH_2_. Subsequently, methanogenesis, the final step, occurs in a separate reactor using the spent medium from the first stage to generate bioCH_4_ (Shanmugam et al. 2021a).

A dual-stage system for biohythane production offers several advantages over a single-stage system. Firstly, separating bioH_2_ and bioCH_4_ production into two distinct stages enhances process stability (Chu et al. 2010; Sasidhar et al. 2022). Each stage can be controlled independently, optimizing environmental conditions and microbial activities. This separation reduces potential inhibitory effects between acidogenic and methanogenic bacteria, minimizing process disruptions and improving stability. Secondly, the two-stage process shortens fermentation time and allows for a higher organic loading rate, increasing biohythane production efficiency (Ueno et al. 2007b; Rajendran et al. 2020). In the initial stage, acidogenic bacteria rapidly convert organic matter into VFAs, accelerating the breakdown of complex organic compounds. This shorter first stage enables earlier commencement of the methanogenesis stage, leading to a quicker overall fermentation period. Additionally, the rapid conversion of organic materials in the first stage allows for a higher feedstock input, facilitating an increased organic loading rate for improved productivity. Thirdly, by leveraging the unique advantages of each stage and optimizing the two-stage AD system, energy recovery from organic matter can be significantly enhanced (Kvesitadze et al. 2012; Hans and Kumar 2019). These benefits make the two-stage system a more efficient and sustainable approach to biohythane production. Table 3 summarizes the advantages and disadvantages of both single-stage and two-stage AD.

**Table 3:** Advantages and disadvantages of single-stage and two-stage AD

	Single-stage AD	Two-stage AD	Ref.
Advantages	- Ease of scale-up - Simplified operation with fewer stages - Lower initial investment - Lower operational costs	- Independent control of each stage - Greater process stability - Reduced fermentation time - Increased organic loading rate - Higher energy recovery - Higher efficiency due to minimized process disruptions, shorter overall fermentation period, and increased productivity	Ueno et al. (2007b); Chu et al. (2010); Kvesitadze et al. (2012); Hans and Kumar (2019); Rajendran et al. (2020); Sasidhar et al. (2022)
Disadvantages	- Higher risk of process disruptions due to potential inhibitory effects - Less flexibility in optimizing conditions - Longer fermentation period - Limited organic loading rate - Lower energy recovery - Lower efficiency due to potential process disruptions and longer overall duration	- More complex system design and operation - Higher initial capital investment - Higher operational costs	

## BioH_2_ and bioCH_4_ production pathways

The initial stages of AD, including hydrolysis, acidogenesis, and acetogenesis, exhibit similarities to dark fermentation (DF) for generating bioH_2_ gas. In the DF process utilizing glucose as the substrate, hydrogen-producing bacteria metabolize glucose into pyruvate through glycolytic pathways. During the conversion process, the system produces adenosine triphosphate (ATP), the main energy carrier in cells, from adenosine diphosphate (ADP), a precursor molecule for energy production, and nicotinamide adenine dinucleotide (NADH) in its reduced form, which is essential for electron transport (Ma et al. 2015; Srivastava et al. 2021). Equation 1 illustrates the specific details of this biochemical pathway. Obligate anaerobes then convert pyruvate into acetyl coenzyme A (acetyl-CoA) using the enzyme pyruvate-ferredoxin oxidoreductase (PFOR) and ferredoxin (Fd), leading to the production of H_2_ by hydrogenase (Eqs. (2) and (3)) (Ma et al. 2015). In contrast, facultative anaerobes utilize pyruvate formate lyase (PFL) to oxidize pyruvate to acetyl-CoA and formate, which is subsequently cleaved into CO_2_ and H_2_ by formate hydrogen lyase (FHL) as described in Eqs. (4) and (5) (Ntaikou et al. 2010; Liu et al. 2017).

1 $${\mathrm{C}}_6{\mathrm{H}}_{12}{\mathrm{O}}_6+2\ \mathrm{NA}{\mathrm{D}}^{+}+2\ \mathrm{ADP}+2\ {\mathrm{P}}_{\mathrm{inorganic}}\to 2\ \mathrm{Pyruvate}+2\ \mathrm{NA}\mathrm{DH}+2\ {\mathrm{H}}^{+}+2\ \mathrm{ATP}$$

2 $$\mathrm{Pyruvate}+\mathrm{C}\mathrm{oA}+2\mathrm{Fd}\ \left(\mathrm{ox}\right)\to \mathrm{Acetyl}-\mathrm{CoA}+2\mathrm{Fd}\ \left(\mathrm{red}\right)+\mathrm{C}{\mathrm{O}}_2$$

3 $$2{\mathrm{H}}^{+}+\mathrm{Fd}\ \left(\mathrm{red}\right)\to {\mathrm{H}}_2+\mathrm{Fd}\ \left(\mathrm{ox}\right)$$

4 $$\mathrm{Pyruvate}+\mathrm{CoA}\to \mathrm{Acetyl}-\mathrm{CoA}+\mathrm{Formate}$$

5 $$\mathrm{Formate}\to \mathrm{C}{\mathrm{O}}_2+{\mathrm{H}}_2$$

The quantity of H_2_ produced depends on the final product of pyruvate oxidation. When acetate is the exclusive end product, four moles of H_2_ are generated per mole of glucose. In contrast, if the end product is butyrate, only two moles of H_2_ are formed (Eqs. (6) and (7)) (Alavi-Borazjani et al. 2019; Chozhavendhan et al. 2020).

6 $${\mathrm{C}}_6{\mathrm{H}}_{12}{\mathrm{O}}_6+2{\mathrm{H}}_2\mathrm{O}\to 2\mathrm{C}{\mathrm{H}}_3\mathrm{COOH}+2\mathrm{C}{\mathrm{O}}_2+4{\mathrm{H}}_2$$

7 $${\mathrm{C}}_6{\mathrm{H}}_{12}{\mathrm{O}}_6+2{\mathrm{H}}_2\mathrm{O}\to 2\mathrm{C}{\mathrm{H}}_3\mathrm{C}{\mathrm{H}}_2\mathrm{C}{\mathrm{H}}_2\mathrm{C}\mathrm{OOH}+2\mathrm{C}{\mathrm{O}}_2+2{\mathrm{H}}_2$$

**Fig. 4:**
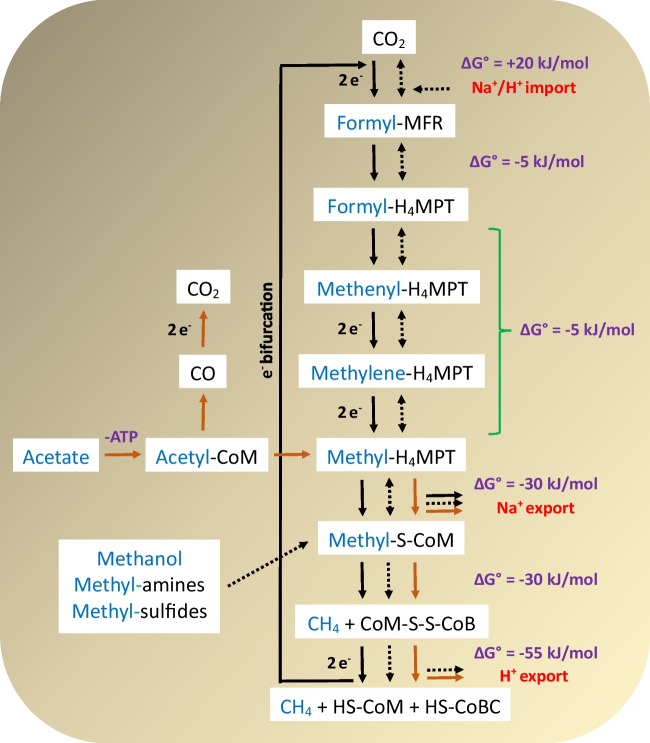
Pathways of methanogenesis (adapted and redrawn from Costa and Leigh (2014)). Solid black, dashed black, and solid brown lines depict hydrogenotrophic, methylotrophic, and acetoclastic reactions, respectively

There are three primary pathways for methanogenesis: (1) hydrogenotrophic, (2) acetoclastic, and (3) methylotrophic (Chen et al. 2022). In hydrogenotrophic methanogenesis, CO_2_ is reduced to CH_4_ using H_2_ or formate as electron donors (Ferry 2011). The reduced ferredoxin plays a crucial role in the initial step of methanogenesis, where CO_2_ is reduced to a formyl group attached to the methanofuran (MFR) carrier molecule. The formyl group is subsequently moved to the tetrahydromethanopterin (H_4_MPT) carrier. This transfer induces cyclization through dehydration, resulting in the formation of methenyl-H_4_MPT. The methenyl group undergoes a two-step reduction process, initially forming a methylene and subsequently a methyl group. The resulting methyl group is then attached to coenzyme M (HS-CoM), which contains a sulfhydryl group. Finally, coenzyme B (HS-CoB) oxidizes HS-CoM, leading to the formation of CH_4_ and a heterodisulfide intermediate (CoM-S-S-CoB). This heterodisulfide undergoes further reduction to restore HS-CoM and HS-CoB (Costa and Leigh 2014). In hydrogenotrophic methanogenesis, a key methyl transfer reaction within the core pathway is directly linked to energy capture. The coenzyme M methyltransferase (Mtr) translocates sodium ions across the membrane, creating a sodium driving force that is utilized by an ATP synthase (Kurth et al. 2020). In acetoclastic methanogenesis, acetate is transported into microbial cells and converted to acetyl-CoA through acetate kinase/phosphotransacetylase or acetyl-CoA synthetase (Berger et al. 2012). The acetyl-CoA decarbonylase/synthase complex then cleaves the acetyl group in a dismutation reaction. This reaction generates CO_2_ and channels the remaining methyl group toward the central methanogenic pathway for conversion to CH_4_ (Kurth et al. 2020). Energy conservation in this process involves membrane-bound methyltransferase Mtr and a membrane-bound electron transport chain utilizing reduced ferredoxin and heterodisulfide (Welte and Deppenmeier 2014). Acetoclastic methanogenesis transports more sodium/proton ions per cycle compared to hydrogenotrophic methanogenesis but requires an initial ATP investment during acetate activation. In methylotrophic methanogenesis, monocarbon compounds like methanol, methylamines, and methyl sulfides function as both electron donors and acceptors (Söllinger and Urich 2019). The pathway begins with these substrates entering as methyl-S-CoM. The reduction of methyl-S-CoM to CH_4_ involves transferring electrons either from H_2_ or through oxidizing another molecule of methyl-S-CoM to CO_2_ in a process known as methyl disproportionation (Costa and Leigh 2014). Three-quarters of the methyl groups are reduced to CH_4_ in methylotrophic methanogenesis, with the remaining quarter oxidized to CO_2_. Energy conservation occurs through membrane-bound electron transport, with membrane-bound methyltransferase functioning in the reverse reaction, dissipating the proton/sodium driving force (Kurth et al. 2020). A common step across all methanogenesis pathways is the reduction of methyl-S-CoM and the breakdown of CoM-S-S-CoB. Figure 4 provides a simplified illustration of the three methane production pathways.

## Microbial diversity in biohythane production

The generation of bioH_2_ and bioCH_4_ through the AD process is influenced by an intricate interplay of various microorganisms, each having distinct environmental needs. To attain the targeted biohythane makeup with the ideal bioH_2_/bioCH_4_ ratio, precise management of the growth of these microorganisms according to their individual traits is essential. More information regarding the particular microorganisms participating in both hydrogenogenic and methanogenic processes is outlined below.

### Microorganisms in bioH_2_ production process

Various microorganisms play a role in bioH_2_ production, including strict anaerobes, facultative anaerobes, and even certain aerobes (Ghimire et al. 2015). *Clostridia* are the primary bioH_2_-producing microorganisms under mesophilic conditions (Fang et al. 2002). These spore-forming obligate anaerobes have a shorter doubling time and greater resilience under unfavorable conditions compared to other anaerobic bacteria, making them ideal for industrial applications (Roy and Das 2016). Additionally, *Clostridia* can degrade crystalline cellulose, a highly resistant substrate (Wei et al. 2014). *Enterobacter*, a non-sporulating facultative anaerobe with a faster growth rate than obligate anaerobes, is another significant bioH_2_ producer (Hans and Kumar 2019). *Enterobacter* bacteria can utilize various carbon sources as substrates, but there are metabolic differences between them and *Clostridium* sp., especially in the fermentation by-products (Tapia-Venegas et al. 2015). Studies have shown that *Enterobacter* sp. has a lower bioH_2_ production yield compared to *Clostridium* sp. (Abdur Rawoof et al. 2021) and is more sensitive to traces of dissolved oxygen (Roy and Das 2016). Within the *Bacillus* genus, which are typically facultative anaerobes, robust H_2_-producing bacteria like *Bacillus licheniformis* and *Bacillus coagulans* have been identified. Their ability to thrive in the presence of dissolved oxygen makes them attractive for industrial applications compared to strict anaerobes. *E. coli* has been widely used for genetic manipulation to enhance bioH_2_ production. For example, overexpression of formate hydrogen lyase (FHL) in *E. coli* increased bioH_2_ formation by 2.5-fold (Yoshida et al. 2005). Similarly, modifying the metabolic pathways of *E. coli* by selectively removing certain genes resulted in a fivefold enhancement in bioH_2_ production (Tran et al. 2014). In thermophilic dark fermentation, *Thermoanaerobacter* sp., *Thermoanaerobacterium* sp., and *Clostridium* sp. are the dominant bacterial species responsible for bioH_2_ production (Mozhiarasi et al. 2023). *Caldicellulosiruptor* sp. and *Thermotoga* sp. are examples of obligate fermentative anaerobes known for their bioH_2_ production under extreme thermophilic conditions (Brynjarsdottir et al. 2013).

In dark fermentation systems, various microorganisms play a role in enhancing bioH_2_ production, even though they do not directly produce bioH_2_. For instance, *Streptococcus* sp. has been reported to enhance bioH_2_ production through granule formation (Hung et al. 2011a). Similarly, *Bifidobacterium* sp. aids in biohydrogenation by decomposing complex organic compounds into smaller molecules suitable for uptake by hydrogen-producing bacteria (Cheng et al. 2008a; Doi et al. 2009). Studies have also shown that the presence of *Klebsiella* sp. in the fermentation medium can create anaerobic conditions by removing oxygen, which is advantageous for obligate bioH_2_-producing bacteria like *Clostridium* sp. (Hung et al. 2011b)*.* Table 4 provides a list of significant microorganisms involved in bioH_2_ production, including both direct H_2_ producers and those that facilitate hydrogen generation, along with their key characteristics.

**Table 4:** Microorganisms involved in biohydrogen production

Genera	Order	Characteristics	Ref.
*Clostridium*	*Eubacteriales*	- Strict anaerobes - Mesophilic and thermophilic conditions - Spore-forming - Short doubling time - Greater resilience under unfavorable conditions - Ability to degrade crystalline cellulose	Fang et al. (2002); Wei et al. (2014); Roy and Das (2016); Mozhiarasi et al. (2023)
*Enterobacter*	*Enterobacterales*	- Facultative anaerobes - Mesophilic conditions - Non-sporulating - Faster growth rate than obligate anaerobes - Ability to utilize various carbon sources - Lower bioH_2_ production compared to *Clostridium* sp. - Vulnerability to traces of dissolved oxygen	Tapia-Venegas et al. (2015); Roy and Das (2016); Hans and Kumar (2019); Abdur Rawoof et al. (2021)
*Bacillus*	*Bacillales*	- Facultative anaerobes - Mesophilic conditions - Industrially important - Ease of use - Metabolically versatile - Rapid growth rate - Survivability in the presence of dissolved oxygen - Retaining an anaerobic environment through oxygen removal - Ability to produce bioactive molecules such as acyl-homoserine lactonases - Capability to secrete extracellular proteins - Minimal mineral salt requirement	Chang et al. (2008); Hung et al. 2011a; Song et al. (2013); Kumar et al. (2015); Abubackar et al. (2019)
*E. coli*	*Enterobacteriales*	- Facultative anaerobes - Non-sporulating - Mesophilic conditions - Well-defined in physiological and biochemical aspects - Extensively utilized for genetic manipulation - Simple cultivation and storage - High growth rate	Yoshida et al. (2005); Redwood et al. (2008); Maeda et al. (2012); Tran et al. (2014)
*Thermoanaerobacter*	*Thermoanaerobacterales*	- Strict anaerobes - Thermophilic conditions - Short generation time - Resilience to harsh environmental conditions - Ability to use a wide range of substrates - Enhanced bioH_2_ formation with few by-products	Singh et al. (2014); Vipotnik et al. (2016); Mozhiarasi et al. (2023)
*Thermoanaerobacterium*	*Thermoanaerobacterales*	- Strict anaerobes - Thermophilic conditions - Ability to use a broad substrate spectrum specially polysaccharide and carbohydrate - Increased bioH_2_ production with minimal by-products	O-Thong et al. (2017); Litti et al. (2022); Mozhiarasi et al. (2023)
*Caldicellulosiruptor*	*Thermoanaerobacterales*	- Strict anaerobes - Extreme thermophilic conditions - Ability to utilize a broad range of carbohydrates - Efficient degradation of recalcitrant substrates - Relatively simple growth requirements - BioH_2_ yields close to the theoretical maximum	Zeidan and van Niel (2010); Bielen et al. (2013); Brynjarsdottir et al. (2013)
*Thermotoga*	*Thermotogae*	- Strict anaerobes - Extreme thermophilic conditions - Capability to utilize a wide range of substrates - BioH_2_ yields nearing the theoretical maximum - Limitation in the degradation of cellulosic compounds	Brynjarsdottir et al. (2013); Pradhan et al. (2015); Shao et al. (2020)
*Streptococcus*	*Lactobacillales*	- Facultative anaerobes - Mesophilic conditions - Effective in granular formation - Maintenance of anaerobiosis in the system	Hung et al. (2011a); Perna et al. (2013); Pugazhendhi et al. (2019)
*Bifidobacterium*	*Bifidobacteriales*	- Anaerobes - Mesophilic conditions - Breaking down complex organic substrates - Low acid tolerance	(Cheng et al. (2008a); Doi et al. (2009); Gomez-Romero et al. (2014); Cieciura-Włoch et al. (2020)
*Klebsiella*	*Enterobacteriales*	- Facultative anaerobes - Mesophilic conditions - Rapid growth rate - Simple growth condition - Creating anaerobic conditions - Capable of converting crude glycerol into bioH_2_	Niu et al. (2010); Hung et al. (2011b); Chookaew et al. (2014)

**Fig. 5:**
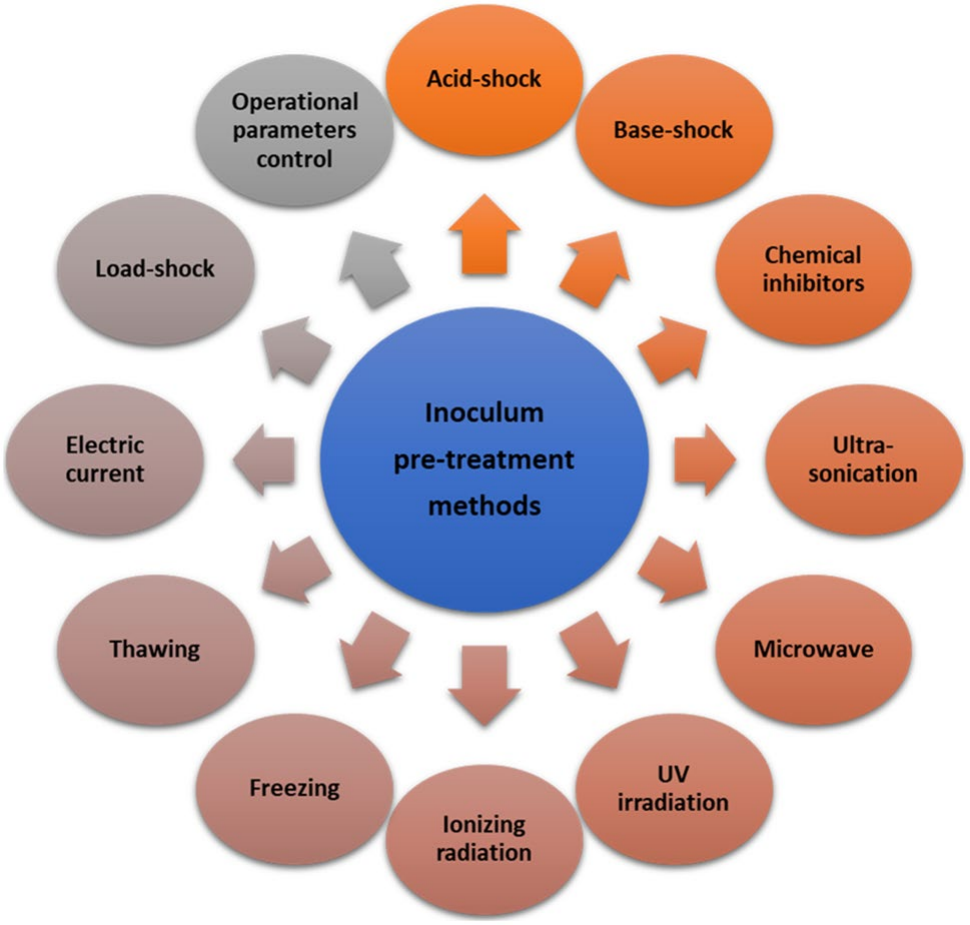
Inoculum pre-treatment methods for enrichment of hydrogen-producing bacteria

It is important to understand that the roles of coexisting microorganisms in bioH_2_ production can be intricate and dependent on the context. For instance, *Bacillus* sp. has been found to have both positive and negative impacts on bioH_2_ production in various fermentation systems (Hung et al. 2011a). Therefore, the influence of coexisting microorganisms on bioH_2_ formation is not always straightforward and requires careful consideration. Additionally, when dealing with mixed cultures, there is a possibility of undesired microorganisms coexisting with bioH_2_ producers, which can hinder bioH_2_ formation or consume bioH_2_ (Tapia-Venegas et al. 2015). However, this issue can be addressed by treating the inoculum before use. Figure 5 depicts different pre-treatment techniques for enhancing the population of bioH_2_-producing bacteria. Among these methods, heat shock treatment is the most commonly utilized. This method involves subjecting the sample to high temperatures for a specific duration, followed by a gradual cooling process to return it to room temperature. Previous studies have shown that optimal temperature and duration for pre-treatment can vary depending on the inoculum source. Reported temperatures range from 65 °C (Baghchehsaraee et al. 2011) to 121 °C (Wang et al. 2003), with treatment durations as short as 10 min (Cavalcante de Amorim et al. 2009) and as long as 5 h (Argun et al. 2008). Another common pre-treatment strategy for dark fermentative hydrogen production utilizes acid or base shocks to selectively eliminate unwanted microorganisms, especially methanogens, from the seed sludge. According to existing literature, acid-shock treatment typically involves a pH range of 2 (De Sá et al. 2013) to 4 (Sen and Suttar 2012) and exposure duration of 30 min (Zhu and Béland 2006) to 24 h (Elbeshbishy et al. 2010). Similarly, base-shock treatment typically utilized pH levels between 10 and 12 and exposure times ranging from 30 min (Zhu and Béland 2006) to 24 h (Kan 2013). The effectiveness of acid/base-shock treatment for enriching hydrogen producers remains a topic of debate, as existing literature does not provide consistent conclusions. While some studies have shown better results with acid-shock treatment (Chang et al. 2011), others have found base-shock treatment to be more effective (Zhu and Béland 2006; Wang and Wan 2008a; Yin et al. 2014). Moreover, while some studies have indicated that pH treatment can lead to higher bioH_2_ yield compared to heat treatment, the general consensus is that heat treatment is a more effective method for enhancing the bioH_2_ production capacity of the inoculum (Wang and Yin 2017).

### Microorganisms in bioCH_4_ production process

Archaea, specifically those within the Euryarchaeota phylum, are the primary microorganisms responsible for methanogenesis. These microorganisms have distinct characteristics that set them apart from bacteria and eukaryotes (Roy and Das 2016; He et al. 2019). They are highly sensitive to oxygen and can only thrive in completely anoxic environments (DasSarma et al. 2009). According to a recent taxonomic proposal, methanogens can be categorized into six orders, 13 families, and 33 genera (Kim and Whitman 2014). Methanogens can also be classified into three categories based on the pathway of bioCH_4_ formation: hydrogenotrophs, aceticlastics, and methylotrophs. Hydrogenotrophs reduce CO_2_ to CH_4_, while aceticlastics are responsible for converting acetate to CH_4_. Methylotrophs, on the other hand, convert methylated C1 substrates like methanol, methylmercaptopropionate, methylamines, and dimethylsulfide to CH_4_ (Angelidaki et al. 2011).

Most methanogens utilize H_2_ as both an electron donor and energy source to convert CO_2_ into CH_4_ through hydrogenotrophic activity. It is interesting to note that approximately 45% of hydrogenotrophs have the ability to use formate as an alternative to H_2_ (Megonigal et al. 2014). Some hydrogenotrophs from the order *Methanomicrobiales*, such as *Methanoculleus* sp. and *Methanogenium* sp., can function as mixotrophs using a combination of carbon and energy sources (Angelidaki et al. 2011). Acetate oxidation accounts for about two-thirds of the CH_4_ produced in anaerobic systems (Pavlostathis 2011), with only two genera from the *Methanosarcinales* order, *Methanosaeta*, and *Methanosarcina*, involved in acetoclastic methanogenesis (Welte and Deppenmeier 2014). *Methanosaeta* is an obligate acetate-consuming methanogen that thrives in environments with low acetate concentrations, while *Methanosarcina* is versatile and capable of utilizing various carbon sources besides acetate (Kurade et al. 2019). Methylotrophic methanogenesis is primarily found in the *Methanosarcinales* order, with the exception of *Methanosphaera*, a genus from the *Methanobacteriales* order (Conrad 2020).

Most of the known methanogens are either mesophilic or moderately to extremely thermophilic, with only a few species able to survive and produce CH_4_ at low temperatures (Simankova et al. 2003; Angelidaki et al. 2011). The order *Methanobacteriales* consists of two families, *Methanobacteriaceae* and *Methanothermaceae*. While members of the *Methanobacteriaceae* family do not thrive at temperatures above 70 °C, the *Methanothermobacter* genus within this family is prevalent in mildly warm environments and thrives best around 65 °C. In contrast, all members of the *Methanothermaceae* family have an optimal growth temperature range of 83 to 88 °C. The *Methanococcales* and *Methanopyrales* orders also include species that excel at temperatures exceeding 70 °C. For example, *Methanocaldococcus* and *Methanotorris*, two genera within the *Methanococcales* order, can flourish at temperatures ranging from 80 to 88 °C (Topçuoglu and Holden 2019). *Methanopyrus kandleri*, an exceptionally heat-tolerant strain from the *Methanopyrales* order, can even survive at 122 °C. On the other hand, *Methanogenium frigidum* from the *Methanomicrobiales* order is a psychrophilic strain that is highly cold-resistant, capable of thriving between 0 and 17 °C with an optimal temperature of 15 °C (Lyu et al. 2018). Building on the discussion of specific methanogens in this section, Table 5 summarizes their taxonomic classification (order) and key characteristics for easy reference.

**Table 5:** Microorganisms involved in biomethane production

Genera	Order	Characteristics	Ref.
*Methanoculleus*	*Methanomicrobiales*	- Irregular coccoid - Hydrogenotroph - Ability to grow as a mixotroph - Mesophilic to thermophilic conditions (optimum: 20–60 °C) - Slow growth rates - High nutritional requirements - Low NaCl requirements - High ammonia tolerance	Garcia et al. (2006); Whitman et al. (2006); Cheng et al. (2008b); Angelidaki et al. (2011); Shimizu et al. (2013); Oren (2014a); Manzoor et al. (2016)
*Methanogenium*	*Methanomicrobiales*	- Irregular coccoid (greatest diversity) - Hydrogenotroph - Ability to grow as a mixotroph - Psychrophilic to thermophilic conditions (optimum: 15–35 °C) - High nutritional requirements - Low NaCl requirements - Slow growth rate	Ollivier et al. (1986); Garcia et al. (2006); Whitman et al. (2006); Angelidaki et al. (2011); Oren (2014a)
*Methanothermobacter*	*Methanobacteriales*	- Rod-shaped - Hydrogenotroph - Thermophilic conditions (optimum: 55–65 °C) - Readily grow in minimal salt media - Exothermic methanogenic process - Success on industrial scales	Bonin and Boone (2006); Li et al. (2017); Casini et al. (2023)
*Methanosphaera*	*Methanobacteriales*	- Irregular cocci arranged as single cells, in pairs, or most commonly in tetrads - Methylotroph - Grow exclusively by H_2_ and methanol - Mesophilic conditions (optimum: 30–40 °C)	(Bonin and Boone (2006); Whitman et al. (2006); Angelidaki et al. (2011); Mohammadzadeh et al. (2022)
*Methanosarcina*	*Methanosarcinales*	- Coccoid and pseudosarcinal cells - Unique among methanogens in terms of metabolic, physiological, and environmental diversity - Aceticlastic - The broadest substrate spectrum - Containing mildly halotolerant and mildly halophilic species - Mesophilic to thermophilic conditions (optimum: 25–50 °C) - Often predominant at lower pH values - High growth rate - Tendency to grow as irregular clumps of cells	Anderson et al. (2003); Kendall and Boone (2006); Whitman et al. (2006); Angelidaki et al. (2011); Buan et al. (2011); Welte and Deppenmeier (2014)
*Methanosaeta*	*Methanosarcinales*	- Large sheathed rods - Obligate aceticlastic - Mesophilic to thermophilic conditions (optimum: 35–60 °C) - Low growth rate (1–3 days) - Sensitive to pH and ammonia concentration - Challenging to obtain pure cultures	Anderson et al. (2003); Kendall and Boone (2006); Whitman et al. (2006); Angelidaki et al. (2011); Welte and Deppenmeier (2014)
*Methanocaldococcus*	*Methanococcales*	- Coccoid morphology with flagellar tufts - Autotrophic growth - Hydrogenotroph - Hyperthermophilic conditions (optimum: 80–85 °C) - NaCl requirement for optimal growth - Growth stimulation with Selenium and tungsten	Garcia et al. (2006); Whitman and Jeanthon (2006); Oren (2014b)
*Methanotorris*	*Methanococcales*	- Irregular coccus - Autotrophic growth - Hydrogenotroph - Hyperthermophilic conditions (optimum: 75–88 °C)	Whitman and Jeanthon (2006); Oren (2014b)
*Methanopyrus*	*Methanopyrales*	- Short rod cell morphology - Hydrogenotroph - Hyperthermophilic conditions (optimum: 98 °C) - Ability to thrive in highly saline environments	Kurr et al. (1991); Forterre (2006); Garcia et al. (2006); Angelidaki et al. (2011); Oren (2014c)
*Methanocella*	*Methanocellales*	- Rod-shaped, coccoid - Hydrogenotroph - Mesophilic to thermophilic conditions (optimum: 35–55 °C) - Activity preference at extremely low H_2_ levels - Exceptional aero-tolerant abilities - Difficult to cultivate	Sakai et al. (2008, 2014); Lü and Lu (2012); Lyu and Lu (2015)

## Key parameters influencing biohythane production

Various factors influence the generation of bioH_2_ and bioCH_4_ as components of biohythane during AD. Achieving a balance among these factors is crucial for obtaining high-quality biohythane and preventing process failures. While optimizing process parameters for different microbial communities in single-stage AD can be challenging, two-stage AD offers ideal conditions for microbial growth, resulting in maximum efficiency in biohythane production. Key process parameters influencing biohythane production are outlined below.

### pH

pH is considered the most sensitive biochemical factor, influencing the enzymatic machinery of microorganisms and maintaining cell redox potential in AD (Yang et al. 2015; Vongvichiankul et al. 2017). A significant issue in the dark fermentation process is the continuous formation of volatile fatty acids (VFAs), resulting in a significant drop in pH levels and disruption of microbial membranes (Yeshanew et al. 2016; Khan et al. 2018; Kumar et al. 2021). The ideal pH for bioH_2_ generation is typically within a range of 6.0 to 8.0 (Sinha and Pandey 2011), although some strains can produce bioH_2_ even in acidic environments below pH 6.0 (Pandey et al. 2009). At pH levels below 4.5, a metabolic shift from acidogenesis to solventogenesis can occur, negatively impacting bioH_2_ formation (Khanal et al. 2004; Van Ginkel and Logan 2005). BioH_2_ production may cease completely at pH levels of 3.8–4.2 (Shanmugam et al. 2021b; Ghosh and Kar 2022).

For bioCH_4_ production, a pH range of 6.7–7.5 is recommended for optimal methanogen activity (Alavi-Borazjani et al. 2020; Jadhav et al. 2021), as methanogens struggle to thrive below pH 6.5 and above pH 9 (Kabir et al. 2022). Incomplete breakdown of organic matter during the acidogenesis stage disrupts the subsequent methanogenesis stage. This inefficiency can result in the build-up of VFAs and other organic acids, as the microbial populations responsible for converting these intermediates into methane are either not functioning optimally or are overwhelmed. The accumulation of these organic acids results in an increased concentration of hydrogen ions (H^+^), leading to a reduction in pH. This acidic environment further impairs methanogenic activity, creating a vicious cycle. As acidification progresses, more acidic ions dissociate, accelerating the decline in pH. If left unchecked, this cycle can ultimately halt biological methane production (van Lier et al. 2020; Sasidhar et al. 2022).

Maintaining optimal pH levels in AD processes has traditionally relied on the use of chemicals like sodium hydroxide (NaOH), potassium hydroxide (KOH), and sodium carbonate (Na_2_CO_3_) (Xie et al. 2014). However, a more cost-effective approach has emerged with the development of digestate recirculation systems. These systems strategically reintroduce digestate, a nutrient-rich by-product of the methanogenic stage, back into the acidogenic reactor (Aslanzadeh et al. 2013; Algapani et al. 2019; Liu et al. 2019). This recirculation also accelerates substrate degradation, allowing for higher organic loading rates and reducing the required reactor volume (Zuo et al. 2013). However, excessive recirculation can lead to ammonia build-up, which can inhibit both hydrogenogenic and methanogenic processes (Cavinato et al. 2012; Wu et al. 2018). Conversely, very low recirculation ratios may not provide enough buffering capacity to control pH levels effectively (Bolzonella et al. 2018).

### Temperature

Temperature plays a crucial role in the success of two-stage AD for biohythane production. It significantly influences various aspects of the process, including the properties of microbial cells, the types of metabolic by-products generated, and the specific nutrient requirements of the microbes involved (Kumar et al. 2021). Enzymes have an optimal temperature range for their activity, and any deviation from this range can result in denaturation or enzyme inactivation, hindering the process and halting metabolic product production (Shanmugam et al. 2021b). Enzyme activity typically increases by up to twofold for every 1 °C temperature rise until reaching the optimum temperature. However, surpassing the optimal temperature leads to a decline in enzyme activity (Roy and Das 2016). Therefore, optimizing the operational temperature is crucial for improving bioH_2_ and bioCH_4_ production processes.

Microorganisms involved in bioH_2_ production exhibit a remarkable range of optimal growth temperatures. Psychrophiles, for example, thrive in cold environments below 25 °C. Mesophiles function best in moderate temperatures ranging from 25 to 45 °C. Thermophiles, on the other hand, prefer warmer conditions between 45 and 65 °C. Finally, extreme thermophiles demonstrate optimal activity at high temperatures, between 65 and 80 °C. While hyperthermophiles exist and can tolerate temperatures exceeding 80 °C, their extreme temperature requirements make them less commonly employed in bioH_2_ production processes (Alavi-Borazjani et al. 2021a, 2021b). Thermophilic conditions (typically around 55 °C) offer a significant advantage for bioH2 production. This stems from the temperature dependence of hydrogenase, the key enzyme responsible for bioH_2_ generation. Hydrogenase exhibits its peak activity at these elevated temperatures, maximizing the efficiency of the bioH_2_ production process (Reith et al. 2003). As previously stated, creating thermophilic conditions offers an additional benefit in bioH_2_ production by selectively promoting the growth of bioH_2_ producers while inhibiting the growth of competing microorganisms. This is due to the fact that most bioH_2_ producers can form spores and survive in harsh conditions like high temperatures, whereas many unwanted hydrogen-consuming microorganisms are sensitive to high temperatures and can be deactivated (Liu et al. 2018). Increasing operational temperatures has been demonstrated to enhance the breakdown of stubborn organic matter like lipids (Chipasa and Mȩdrzycka 2006). Additionally, elevated temperatures can lead to an increase in reactor pH, which can significantly enhance microbial activity in the bioH_2_ production process (Ventura et al. 2014).

Thermodynamic principles can help explain the impact of temperature on bioH_2_ production. Specifically, the standard enthalpy change (Δ*H*°) during the conversion of glucose to acetate can be examined, assuming a theoretical yield of 4 moles of H_2_ per mole of glucose. This reaction is endothermic (Δ*H*° > 0), meaning it requires an input of energy to proceed. In this case, energy is absorbed in the form of heat, and the products have a higher enthalpy than the reactants (Sarangi and Nanda 2020). The Van’t Hoff equation (Eq. (8)) (Smith et al. 2018), which describes the relationship between temperature and the equilibrium constant (*K*) for a chemical reaction, can also be used to elucidate the thermodynamics of bioH_2_ production:

8 $$\ln \left({K}_2/{K}_1\right)=\left({\Delta \mathrm{H}}^{{}^{\circ}}/R\right)\times \left(1/{T}_1-1/{T}_2\right)$$

where *K*_1_ and *K*_2_ are the equilibrium constants at temperatures *T*_1_ and *T*_2_, respectively, *R* denotes the gas constant, and ln signifies the natural logarithm. If the reaction is exothermic (Δ*H*° < 0), a rise in temperature will result in a decrease in the equilibrium constant, and conversely. This is because an increase in temperature causes the system to shift towards the side of the reaction with the lower heat content to offset the rise in heat energy. For an endothermic reaction (Δ*H*° > 0), the opposite holds true, with a temperature increase leading to an increase in the equilibrium constant. Essentially, at elevated temperatures, the equilibrium constant for the endothermic reaction C_6_H_12_O_6_ + 2H_2_O → 2CH_3_COOH + 2CO_2_ + 4H_2_ will rise, indicating a higher concentration of products compared to reactants. This phenomenon occurs because the endothermic reaction absorbs heat as a reactant, and raising the temperature enhances heat absorption, thereby favoring the forward reaction and H_2_ production.

Methanogenesis exhibits a greater tolerance for temperature fluctuations compared to the hydrogenogenic stage. This is particularly evident under psychrophilic (cold) and mesophilic (moderate) conditions (Gunes et al. 2019; Sasidhar et al. 2022). A slight temperature variation of ± 1 °C can adversely affect bioCH_4_ production in thermophilic conditions, whereas mesophilic methanogens can withstand larger temperature fluctuations of up to ± 3 °C without significant decreases in bioCH_4_ yields (Gunes et al. 2019). Thus, methanogenic reactors can be operated at ambient or mesophilic temperatures without requiring high-energy inputs for heating.

### Retention time

In continuous-mode bioH_2_ and bioCH_4_ production, optimizing retention time within the anaerobic digester is critical. Hydraulic retention time (HRT) refers to the average duration the substrate remains in the digester, while solids retention time (SRT) denotes the average period bacteria are retained in anaerobic digesters (He et al. 2019). HRT is determined by the bioreactor volume, while SRT is influenced by the dominant microbial population in the bioreactor (Sasidhar et al. 2022). In traditional low-rate bioreactors without recycling or supernatant removal, SRT typically equals HRT. However, in high-rate bioreactors utilizing attached or suspended growth mechanisms, SRT is notably longer than HRT (Liu et al. 2011).

Methanogens and other bioH_2_ consumers have a slow growth rate. Therefore, in hydrogenogenic reactors, shorter HRTs can help selectively enrich bioH_2_-producing bacteria and eliminate unwanted microorganisms (Roy and Das 2016). However, a drawback of shorter HRTs is the risk of cell washout, which can lead to decreased bioH_2_ production (Wu et al. 2008). High-rate bioreactors like anaerobic fluidized bed reactors (AFBRs) have been successful in addressing this issue by achieving high biomass concentrations at very low HRTs (Ferreira et al. 2019). Extending the HRT may result in a shift in the bioH_2_ formation pathway towards methanogenesis, potentially inhibiting bioH_2_ production (David et al. 2019). In the methanogenesis stage, a longer HRT is generally recommended compared to the acidogenesis stage. This extended residence time, often around twice that required for slower-growing microbes, allows for several key benefits. First, it ensures sufficient time for the complete metabolization of complex organic compounds into simpler molecules. Second, it provides ample opportunity for the methanogenic microbial population to actively participate in the conversion process, maximizing bioconversion efficiency. Additionally, prolonging the HRT in methanogenic reactors can help eliminate dead zones and promote efficient syntrophy between microorganisms, especially in mixed cultures (Roy and Das 2016).

Overall, there is no set standard for determining the ideal HRT in two-stage bioH_2_ and bioCH_4_ production. It is dependent on several factors, including substrate properties, reactor configuration, process stage, and operating temperature (Kim et al. 2013). Literature shows a wide range of optimal HRT values for the first hydrogenogenic stage, from 3 h (Chu et al. 2022) to 10 days (Wirasembada et al. 2024), and for the second methanogenesis stage, from 3.5 h (Wu et al. 2016b) to 25 days (Dareioti et al. 2022; Sukphun et al. 2023a; Tsigkou et al. 2023).

SRT in two-stage AD processes has not been extensively studied. However, it is generally observed that the SRT for bioH_2_ production is shorter (2–5 days) (Sasidhar et al. 2022) compared to bioCH_4_ production (15–20 days) (Nabaterega et al. 2021). This difference is attributed to the quicker growth rates of hydrogen-producing microorganisms relative to methanogens.

### Organic loading rate

A key factor for a stable and efficient AD process is the organic loading rate (OLR). It reflects the daily organic matter input relative to the digester’s volume (Dangol et al. 2022). Increasing the substrate concentration can raise the OLR and enhance biogas production. However, excessive loading may lead to the accumulation of VFAs and acidification, potentially causing process failure (Grangeiro et al. 2019; Tyagi and Aboudi 2021). Conversely, if the reactor is underfed, it can become overly alkaline, reducing biogas productivity (Periyasamy et al. 2022). Therefore, it is crucial to conduct thorough assessments to determine the optimal OLR for the digester.

The optimal OLR for traditional single-stage AD systems typically falls within the range of 0.5 to 2 kg VS/m^3^/day, influenced by factors like feedstock characteristics, retention time, and operating temperature (Ramanathan et al. 2022). In contrast, two-stage AD systems, which separate the fermentation and methanogenesis phases, provide favorable conditions for distinct microbial communities. Research suggests that these systems can accommodate organic loads up to 300% higher than single-stage AD systems (Rajendran et al. 2020). Additionally, studies have shown that strategically distributing the OLR within a continuous two-stage system can improve biohythane production. This involves allocating a higher OLR to the first stage for bioH_2_ production and a lower OLR to the second stage for bioCH_4_ production (Montiel Corona and Razo-Flores 2018). OLR and HRT are interconnected parameters in anaerobic digestion, with the determination of one parameter dependent on the definition of the other. Consequently, adjusting one parameter can be achieved by fine-tuning the other as necessary (Sasidhar et al. 2022).

### Nutrients

The success of the AD process relies on a balanced supply of macro and micronutrients. These vital nutrients support the complex biochemical activity of microbial communities and their associated enzymatic machinery, which are essential for the process. Nitrogen, phosphorus, and sulfur are essential macronutrients. Adequate nitrogen levels are necessary for microbial protein synthesis as it is a key component of amino acids (Choi et al. 2020). Nitrogen also helps maintain a stable reactive biosystem by providing sufficient buffering capacity (Krakat et al. 2017). However, high nitrogen concentrations in bioreactors can negatively affect process stability and efficiency due to the formation of ammonia, which can disrupt microbial function. Ammonia can enter microbial cells, causing proton imbalance and changes in intracellular pH. Additionally, free ammonia can inhibit specific enzymatic reactions (Morozova et al. 2020). Therefore, high ammonia concentrations can hinder both fermentative bioH_2_ production and methanogenesis processes. While the reported optimal concentration of ammonia for bioH2 production may vary in different studies, a suggested range of 0.01–7 g/L based on available literature data (Bisaillon et al. 2006) can be considered ideal. Studies have shown that ammonia concentrations between 0.05 and 0.2 g/L can have a positive impact on the AD process (McCarty 1964). Critical ammonia concentrations that can lead to methanogenesis inhibition have been reported to range from 1.7 to 14 g/L (Chen et al. 2008b). Optimizing the carbon-to-nitrogen (C/N) ratio within the digester is crucial for both minimizing ammonia concentration and maximizing biogas production. The range of 20 to 30 is widely considered the optimal C/N ratio for the AD process (Yen and Brune 2007; Zhang et al. 2014). Additionally, ensuring adequate phosphate levels is critical for maximizing biohythane production efficiency, as phosphorus is a vital mineral nutrient for microbial growth and function (Lei et al. 2010). Phosphate also plays a vital role in energy transfer and buffering capacity within the biohythane production system, acting as a substitute for carbonate (Chandrasekhar et al. 2015). However, high phosphorus levels, particularly at 3.3 mg/L, can negatively affect methanogenesis by increasing VFA production and lowering pH. Acidogenesis, on the other hand, is less impacted by these conditions (Mancipe-Jiménez et al. 2017). Sulfur is another crucial macronutrient for microorganisms involved in biohythane production, especially methanogens, commonly found in sulfate form in various feedstocks like food processing and pulp and paper waste (Dhar et al. 2012). Elevated sulfate concentrations can hinder dark fermentation and methanogenesis by promoting competition between bioH_2_ and bioCH_4_ producers and sulfate-reducing bacteria for organic matter, as well as causing toxicity from sulfide release (Chen et al. 2008b; Bundhoo and Mohee 2016). The optimal sulfur concentration for bioCH_4_ production in anaerobic digestion is typically 1–25 mg/L (Chen et al. 2008b). Limited information is available on the impact of sulfate concentration on dark fermentation, but some studies suggest that 3000 mg/L sulfate at pH 5.5 may be effective for bioH_2_ production (Lin and Chen 2006; Chen et al. 2008a; Hwang et al. 2009, 2011). High dissolved sulfide concentrations (> 100 mg/L) can inhibit bioH_2_ production during dark fermentation, while concentrations of 100–800 mg/L are inhibitory to methanogens (Chen et al. 2008b; Dhar et al. 2012).

In addition to macronutrients, micronutrients like specific light and heavy metal ions are crucial for the AD process. These micronutrients facilitate the synthesis and function of enzymes and coenzymes essential for bioH_2_ and bioCH_4_ production. These micronutrients also play a vital role in energy and electron transfer as well as microbial cell growth (Shanmugam et al. 2021b). While the right concentrations of light and heavy metals can promote microbial growth, it is crucial to be aware that excessive amounts can have the opposite effect, potentially leading to toxicity or inhibition of microorganisms (Chen et al. 2008b). Furthermore, each micronutrient contributes uniquely to microbial metabolism, and changes in its bioavailability can disrupt this process (Chandrasekhar et al. 2015). Therefore, it is crucial to regulate the levels of these micronutrients carefully to promote optimal microbial growth and avoid any adverse effects. Table 6 provides an overview of the key functions and recommended concentrations of important metal ions in both dark fermentative.

**Table 6:** Main functions and optimal concentrations of essential metals in bioH_2_ and bioCH_4_ production processes

Metals	Functions	Optimum concentrations (mg/L)	
		BioH_2_ production	BioCH_4_ production
Ca	- Cell proliferation and granulation (Yu et al. 2001) - Membrane permeability (Schattauer et al. 2011; Matheri et al. 2016) - Stimulator of bacterial spore germination (Sekoai and Daramola 2018) - Linkage of bacterial cell surface and extracellular polymers (Yu et al. 2001) - Performance enhancer of other metals (Schattauer et al. 2011)	5.4–1000 (Lee et al. 2004; Sekoai and Daramola 2018)	100–1035 (Romero-güiza et al. 2016)
K	- Cell wall permeability (Wu et al. 2016a) - Regulation of cytoplasmic pH and osmotic pressure (Kakinuma and Harold 1985)	NA^a^	< 400 (Romero-güiza et al. 2016)
Mg	- Electron transfer facilitator (Alavi-borazjani et al. 2022) - Present in cellular walls and membranes (Srikanth and Mohan 2012; Bundhoo and Mohee 2016) - Activator of some kinases and phosphotransferases (Burgess et al. 1999; Schattauer et al. 2011) - Cofactor in glycolysis enzymes (Alavi-Borazjani et al. 2022) - Effective in reducing sodium toxicity (Wu et al. 2016a)	50–1000 (Azbar et al. 2009; Sekoai and Daramola 2018)	< 720 (Romero-güiza et al. 2016)
Na	- Necessary for bacterial growth (Bundhoo and Mohee 2016) - Creation of Na^+^ gradient and promotion of the ferredoxin reduction by nicotinamide adenine dinucleotide (NADH) (Bundhoo and Mohee 2016)	270–9830 (Cao and Zhao 2009; Kim et al. 2009)	100–350 (Romero-güiza et al. 2016)
Co	- Metallic enzyme activator (Burgess et al. 1999; Schattauer et al. 2011) - Present in corrinoids (Schattauer et al. 2011) - Present in super dismutase, hydrogenase, and carbon monoxide dehydrogenase (Wu et al. 2016a)	8.7 (Keskin et al. 2018)	0.006–0.12 (Demirel and Scherer 2011); 0.148–0.580 (Lo et al. 2012)
Cu	- Enzyme activator (Burgess et al. 1999) - Chelating other substances and reducing their toxicity (Burgess et al. 1999; Schattauer et al. 2011)	3–400 (Zheng and Yu 2004; Lin and Shei 2008)	0.03–2.4 (Yue et al. 2007; Romero-güiza et al. 2016)
Fe	- Electron acceptor and donor (Thanh et al. 2016) - Synthesis of catalase, aconitase, and peroxidase (Burgess et al. 1999; Schattauer et al. 2011) - Present in ferredoxin hydrogenase, format dehydrogenase, and carbon monoxide dehydrogenase (Schattauer et al. 2011; Wu et al. 2016a) - Redox reactions (Schattauer et al. 2011; Wu et al. 2016a) - Binding agent in sulfide precipitation (Thanh et al. 2016) - Promoter of the extracellular polymers excretion (Wu et al. 2016a)	3–1000 (Lin and Lay 2005; Sekoai and Daramola 2018)	< 0.3 (Romero-güiza et al. 2016); 0.28–50.4 (Demirel and Scherer 2011)
Mo	- Present in format dehydrogenase and formylmethanofuran dehydrogenase (Schattauer et al. 2011) - Inhibitor of sulfate-reducing bacteria (Schattauer et al. 2011; Wu et al. 2016a)	69 (Hakobyan et al. 2012)	0.044–100 (Mao et al. 2015)
Mn	- Interchangeable with Mg in kinase reactions (Schattauer et al. 2011) - Cofactor in some antioxidant enzymes such as superoxide dismutase (Srikanth and Mohan 2012) - Activator of isocitric dehydrogenase and malic enzymes (Burgess et al. 1999; Schattauer et al. 2011) - Stabilizer of methyltransferase (Schattauer et al. 2011; Wu et al. 2016a) - Redox reactions (Srikanth and Mohan 2012)	15–215 (Hakobyan et al. 2012; Srikanth and Mohan 2012)	< 0.027 (Romero-güiza et al. 2016)
Ni	- Present in carbon monoxide dehydrogenase, methyl reductase, and hydrogenases (Schattauer et al. 2011) - Synthesis of factor F_430_, coenzyme A, and CH_3_-CoM reductase (Schattauer et al. 2011; Choong et al. 2016; Wu et al. 2016a; Passaris et al. 2018) - DNA/RNA stabilizer (Schattauer et al. 2011; Wu et al. 2016a) - Essential for sulfate-reducing bacteria (Wu et al. 2016a) - Cofactor of urease (Schattauer et al. 2011; Matheri et al. 2016) - Biomass maintenance (Burgess et al. 1999)	0.1–1000 (Wang and Wan 2008b; Sekoai and Daramola 2018)	0.03–27 (Romero-güiza et al. 2016); 0.012–5 (Demirel and Scherer 2011)
Se	- Present in format dehydrogenase, formylmethanofuran dehydrogenase, and carbon monoxide dehydrogenase (Schattauer et al. 2011; Romero-güiza et al. 2016) - Synthesis of acetyl-coenzyme A (Schattauer et al. 2011)	0.5 (Keskin et al. 2018)	< 0.04 (Romero-güiza et al. 2016); 0.079–0.79 (Demirel and Scherer 2011)
Zn	- Metallic enzyme activator (Burgess et al. 1999; Schattauer et al. 2011) - Cell growth stimulator (Burgess et al. 1999; Schattauer et al. 2011; Matheri et al. 2016) - Cell maintenance (Zhang et al. 2021) - Cofactor of RNA/DNA polymerase (Schattauer et al. 2011) - Present in format dehydrogenase, superoxide dismutase, and hydrogenase (Schattauer et al. 2011; Wu et al. 2016a)	0.1–80 (Cho and Lee 2011; Keskin et al. 2018)	0.03–2 (Romero-Güiza et al. 2016)

^a^NA, not available

## Potential feedstocks for biohythane production

The economic feasibility of the biohythane production process is closely tied to the use of renewable and cost-effective substrates that require minimal pre-treatment. While easily biodegradable simple sugars like glucose, sucrose, and starch have a high potential for biogas production, their expensive purchase price often makes this approach economically inefficient. To overcome this issue, replacing these materials with renewable organic feedstocks, especially biowaste, is widely acknowledged as a cost-efficient and sustainable strategy for biohythane production. By using biowaste as a feedstock, the process can benefit from lower substrate costs and the added bonus of waste management, making it an appealing choice for economic viability and environmental sustainability.

### Organic fraction of municipal solid waste

Studies have extensively explored the organic fraction of municipal solid waste (OFMSW), particularly food waste, as a promising substrate for biohythane production (Sasidhar et al. 2022). The makeup and properties of these wastes differ based on their source and the season they are produced. In winter, food residues typically have higher protein content, while in summer, they are rich in carbohydrates due to reduced meat consumption and increased fruit and vegetable consumption (Bolzonella et al. 2018). Food waste also contains lipids, which produce higher-quality biogas compared to carbohydrates and proteins (Dasa et al. 2016). However, anaerobic digesters may face operational challenges due to lipid-induced clogging (Cirne et al. 2007). The breakdown of lipids leads to the formation of long-chain fatty acids (LCFAs), which can impede mass transfer by binding to microbial cell walls (Rinzema et al. 1994; Rasit et al. 2015). Additionally, fat adhesion can lead to biomass flotation and the loss of active biomass through washout (Hwu et al. 1998).

Fruit and vegetable wastes have a higher C/N ratio compared to meat residues, and their combination with other food waste portions influences the overall C/N ratio of the substrate (Bolzonella et al. 2018). High levels of lignin and cellulose in fruit and vegetable wastes can pose challenges for dark fermentation and methanogenesis, ultimately reducing biohythane yield (Meena et al. 2020). Managing pH levels is a key challenge when treating diverse substrates like food wastes (Kumar et al. 2021).

The range of biohythane production from various substrates in this category is wide, with values ranging from 23.6 mL/g VS for catering waste (Elwakeel et al. 2023) to 911.9 mL/g VS for OFMSW (Alavi-Borazjani et al. 2022). This variability highlights the significant impact of substrate type and operational conditions on biohythane yield. For instance, organic solid waste from a regional market yielded 258 mL/g VS of biohythane (Salazar-Batres et al. 2024), while household solid waste produced 543 mL/g VS (Liu et al. 2006). Catering waste, despite its low value of 23.6 mL/g VS in batch conditions, showed a biohythane production of 120.94 mL/g VS in continuous mode (Elwakeel et al. 2023). The range for OFMSW varies from 343 (Yeshanew et al. 2018) to 911.9 mL/g VS (Alavi-borazjani et al. 2022). Additionally, raw and processed food waste exhibit significant variability. Raw food waste values range from 212.88 (Cheng et al. 2016) to 669 mL/g VS (Chu et al. 2008). Processed food waste examples include food waste hydrolysate at 395.16 mL/g VS (Pomdaeng et al. 2024) and pre-aerated protein-rich food waste at 356.96 mL/g VS (Rafieenia et al. 2017).

### Agricultural residues

Agricultural residues, including stalks, leaves, husks, and other organic materials from crops, serve as valuable and renewable substrates for biohythane generation. Despite their different physical appearances, all types of lignocellulosic biomass have a consistent chemical composition, including cellulose (30–70%), hemicellulose (15–30%), lignin (10–25%), and hydrophilic/lipophilic extractives (Ahmad et al. 2018). These substrates are rich in nitrogen and carbohydrates, making them suitable for maintaining the optimal C/N ratio required for biohythane production (Abdur Rawoof et al. 2021). However, the challenge lies in the rigid three-dimensional structure of these feedstocks, which hinders their biodegradation (Levin et al. 2009). Hence, it is crucial to employ efficient pre-treatment techniques, including physical, chemical, physicochemical, and biological processes, to break down the intricate composition of these feedstocks and enhance biogas production efficiency (Dar et al. 2021).

The biohythane yields from combining bioH_2_ produced during the first stage and bioCH_4_ generated in the second stage of AD processes treating agricultural residues exhibit considerable variation. For instance, tomato plant residues can yield 264 mL of biohythane per gram of VS (Ruiz-Aguilar et al. 2022). Wheat straw, a common agricultural residue, has shown a biohythane production of 396 mL/g VS (Kongjan et al. 2011). Sweet sorghum, as a substrate, has produced 39.4 mL of biohythane per gram of substrate (Antonopoulou et al. 2008). Date fruit wastes are particularly potent, generating 611 mL/g VS of biohythane (Saidi et al. 2023). Moreover, sugarcane straw hemicellulose hydrolyzate has been used to produce 288 mL/g COD of biohythane (Tomasini et al. 2023). These results highlight the significant potential of various agricultural residues in biohythane production, emphasizing their role as sustainable and efficient feedstocks in renewable energy generation.

### Distillery/brewery wastes

Distilleries and breweries produce significant amounts of waste, posing environmental concerns because of the elevated levels of organic matter, nutrients (phosphorus, ammonia), and heavy metals (copper) (Dionisi et al. 2014). However, these waste streams can also be utilized as valuable resources for bioenergy production, especially for biohythane production, given their rich organic content (Roy and Das 2016).

Investigating the potential of distillery/brewery wastes for biohythane recovery, stillage, a common by-product in distilleries, has shown significant promise. Studies have shown that stillage can yield a combined bioH_2_ and bioCH_4_ yield of 417 mL/g VS (Luo et al. 2011). Furthermore, a mixture of stillage and excess sludge, tested under batch conditions, resulted in a biohythane production of 227 mL/g VS, while continuous conditions increased the biohythane production to 413 mL/g VS (Wang et al. 2011). Cassava stillage, derived from the fermentation and distillation processes involving cassava roots, has also shown potential in biohythane generation with a combined bioH_2_ and bioCH_4_ yield of 306 mL/g VS (Luo et al. 2010). Additionally, a combination of vinasse with spent brewer yeast resulted in a biohythane production of 258 mL/g VS (Nualsri et al. 2024).

Despite their rich organic content, distillery and brewery wastes have a complex and varied structure that makes them difficult for microbes to break down. Additionally, certain waste streams like pot ale contain high protein content, which can lead to increased ammonia concentrations within the bioreactor. These elevated ammonia levels have the potential to disrupt or even halt biogas production (Meena et al. 2020). Nevertheless, effective pre-treatment techniques and process adjustments can improve biohythane production from these plentiful waste sources, promoting the advancement of sustainable and eco-friendly bioenergy systems.

### Dairy processing wastes

Dairy processing produces various liquid and solid by-products and wastes. Whey, the main by-product of the dairy industry, presents challenges for disposal because of its high because of its high nitrogen content and levels of biological and chemical oxygen demand (BOD and COD, respectively) (Kozłowski et al. 2019). However, whey is mainly made up of lactose (around 70%), a fermentable carbohydrate that can be turned into biogas (Meena et al. 2020). It also contains lipids, proteins, soluble vitamins, and minerals (Ahmad et al. 2019), making it a valuable resource for bioenergy production.

A recent trend in biofuel research focuses on harnessing these abundant waste materials to generate bioH_2_ and bioCH_4_, the key components of biohythane. For example, Kovalev et al. (2022) demonstrated that two-stage AD of cheese whey yielded 36.86 mL bioH_2_/g COD in the initial stage, with an additional 325 mL bioCH_4_/g COD in the second stage, resulting in a total production of 361.86 mL/g COD. Similarly, Moreno-Andrade et al. (2015) found 1.11 mole bioH_2_/mole lactose during the initial stage and observed 170 mL bioCH_4_/g COD in the subsequent phase. Another two-stage approach achieved a bioH_2_ yield of 0.78 mole /mole glucose_consumed_, followed by the generation of 147 mL of bioCH_4_/g VSS (Venetsaneas et al. 2009). Additionally, de Souza Almeida et al. (2023) conducted a study on biohythane production using a combination of cheese whey and glycerin. They found that in the first stage, bioH_2_ production reached 9.9 mole/kg COD_removed_. In the second stage, bioCH_4_ production was measured at 14 mole/kg COD_removed_, resulting in a total biohythane yield of 23.9 mole/kg COD_removed_.

This in-depth analysis emphasizes the potential of utilizing dairy processing wastes, specifically cheese whey, for biohythane production. However, the use of low-alkaline substrates like cheese whey poses a challenge due to their tendency to undergo rapid acidification, leading to reduced biogas production or process inhibition (Meena et al. 2020). To address this issue, strategies such as incorporating cost-effective buffering additives, integrating alkaline-rich co-substrates, and implementing advanced monitoring systems can help mitigate the negative effects of rapid acidification and ensure the economic viability of bioenergy production from low-alkaline substrates such as cheese whey.

### Livestock wastes

Livestock wastes generated in farms and slaughterhouses contain substantial amounts of protein, lipids, COD, volatile solids (VS), and low carbohydrate content (Sittijunda et al. 2010). The elevated COD level and microbial composition in these wastes make them highly polluting, and their direct disposal can lead to environmental issues (Meena et al. 2020). Recently, livestock waste has been acknowledged as a valuable raw material for producing bioH_2_ and bioCH_4_ because of its plentiful availability and rich organic matter content. However, as mentioned earlier, the significant lipid content in these wastes can cause biomass flotation and subsequent washout (Cirne et al. 2007). Moreover, lipid degradation leads to the formation of glycerol and long-chain fatty acids, which can accumulate and hinder microbial activity (Moukazis et al. 2018). Conversely, inhibitory effects from a high concentration of ammonia may arise due to protein degradation (Cuetos et al. 2008).

To address these challenges and improve biohythane production, these feedstocks are commonly co-digested with other substrates. This strategy not only helps to maintain nutrient balance but also improves the overall performance of the AD process. Several studies have demonstrated the potential of co-digestion in optimizing biohythane recovery from livestock wastes. For example, a combination of cow dung and untreated domestic wastewater sludge in a batch operation achieved a biohythane production of 877 mL/g VS. Similarly, mixing cattle slurry with grass silage produced 288 mL/g VS biohythane in a batch operation (Ning et al. 2023b) and 248 mL/g VS in a continuous feeding mode (Ning et al. 2023a). Furthermore, the mixture of poultry manure, wine vinasse, and sewage sludge in a continuous operation yielded a biohythane production of 431 mL/g VS by combining the bioH_2_ and bioCH_4_ values from the first and second stages of AD (Cheng et al. 2016). Additionally, co-digestion of chicken manure, corn straw, and food waste led to a biohythane yield of 632.52 mL/g VS in a batch process (Liu et al. 2023).

### Vegetable oil processing wastes

The vegetable oil extraction industry produces a substantial amount of by-products that hold potential for biohythane production. However, using this type of feedstock is not always feasible due to the sensitivity of anaerobes to fat-rich substrates and intermediate compounds resulting from their decomposition (Hidalgo and Martín-marroquín 2014). These residues are typically acidic and contain macronutrients like nitrogen and phosphorus, as well as phenolic compounds that are resistant to biodegradation (Abdur Rawoof et al. 2021). These residues are rich in long-chain fatty acids, which can disrupt mass transfer processes at low concentrations due to their interaction with cell membranes (Hidalgo and Martín-Marroquín 2014). However, recent studies have reported microbial adaptations to long-chain fatty acids (Chen et al. 2008b).

Despite these challenges, numerous studies have investigated the biohythane potential of various vegetable oil processing wastes. For example, olive pulp has shown promise in biohythane production, producing 0.13 mole bioH_2_/kg TS and 0.16 L bioCH_4_/kg COD (Koutrouli et al. 2009). Similarly, palm oil mill effluent has demonstrated significant biohythane production capabilities, with a yield of 535 L/kg COD (Krishnan et al. 2014). Additionally, the combination of palm oil mill effluent and concentrated latex wastewater has shown substantial biohythane production potential, with 95.45 mL bioH_2_/g VS and 1.20 mL bioCH_4_/g VS, leading to a total biohythane production of 96.65 mL/g VS (Raketh et al. 2023). These examples highlight the diverse potential of vegetable oil processing wastes in biohythane production, highlighting the versatility and effectiveness of these feedstocks.

### Algal biomass

Algal biomass, found in both unicellular (microalgae) and multicellular (macroalgae) forms, is becoming increasingly popular for biohythane production. Algae have minimal lignin content, making them highly susceptible to microbial degradation (Montingelli et al. 2016). The energy potential of marine biomass, including algae, is estimated to be over 100 EJ per year, surpassing terrestrial biomass (22 EJ per year) and organic wastes (7 EJ per year) (Chynoweth et al. 2001). Furthermore, algae boast a remarkable potential for CO_2_, as the photosynthetic efficiency of aquatic biomass is reported to be 6–8%, approximately four times greater than that of terrestrial biomass (Aresta et al. 2005; Thompson et al. 2019). Additionally, algae cultivation boasts rapid growth rates and the potential for utilization on non-agricultural or marginal lands. They can even thrive using brackish water or wastewater, thereby reducing the need for competition with food crops for freshwater and fertilizers (Levine et al. 2010; Vivekanand et al. 2012). Notably, algal biomass for biogas production can be naturally harvested from eutrophic and degraded water bodies (Dȩbowski et al. 2013).

Multiple studies have highlighted the significant potential of algal biomass for biohythane production. For example, research combining macroalgae (*Laminaria digitata*) and microalgae (*Nannochloropsis oceanica*) achieved a total biohythane yield of 390.4 mL/g VS, encompassing both bioH_2_ and bioCH_4_ (Ding et al. 2016). Similarly, blending microalgae with fecal sludge produced 24.5 mL bioH_2_/g VS and 281 mL bioCH_4_/g VS, yielding 305.5 mL/g VS of biohythane (Khalekuzzaman et al. 2023). *Chlorella* sp. microalgae generated 9 mL bioH_2_/g VS and 311.9 mL bioCH_4_/g VS, resulting in a total of 321 mL/g VS of biohythane (Wu et al. 2023). Moreover, *S.obliquus* algae generated 56 cm^3^/g VS of bioH_2_ and 119 cm^3^/g VS of bioCH_4_, making a total biohythane yield of 175 cm^3^/g VS (Abimbola et al. 2023).

While the mentioned advantages make algal biomass an attractive option for biohythane production, there are also some drawbacks to consider. For example, specific algae strains with a high nitrogen content (low C/N ratio) may lead to the build-up of ammonia and VFAs in the digester, posing a challenge to efficiently harnessing this plentiful natural resource for biogas production (Zhong et al. 2012). However, this issue can be addressed by co-digesting high-protein algal biomass with a carbonaceous substrate to improve biogas production (Chen et al. 2015). Furthermore, biopolymers such as sporopollenin and algaenan in the rigid cell walls of algae can inhibit hydrolytic enzyme activity, thereby reducing biogas production rates (Kabir et al. 2022). Therefore, appropriate pre-treatment methods are essential to break down these biopolymers and enhance biohythane productivity.

### Other feedstocks

In addition to the previously discussed categorized feedstocks, various other substrates have been investigated for their potential in biohythane production. The biohythane production values for these feedstocks vary widely and are influenced by different factors. For instance, sedimented pulp and paper mill waste fiber have shown promise, with a study reporting a combined bioH_2_ and bioCH_4_ production of 376.1 mL/g VS (El-Qelish et al. 2024). Similarly, tofu-processing residue, also known as okara, has exhibited significant biohythane production capabilities. One study reported a biohythane production of 114.4 mL/g VS, comprising 50 mL bioH_2_/g VS and 64.4 mL bioCH_4_/g VS (Ali et al. 2024), while another study demonstrated a higher biohythane yield of 581.42 mL/g VS (Ali et al. 2022).

Residual glycerin, a by-product of biodiesel production, has also shown promise with a biohythane yield of 976 mL/L_medium_ (de Oliveira Faber et al. 2023). Moreover, corn steep liquor, a by-product of corn wet milling, has demonstrated potential in biohythane recovery with a bioH_2_ production of 670 cm^3^/L/day and a bioCH_4_ production of 220 cm^3^/L/day (Stoyancheva et al. 2023). Halophytic biomass, such as *Atriplex crassifolia*, has also been researched, yielding 13.2 mL bioH_2_/g in the initial stage and 8.5 mL bioCH_4_/mL in the subsequent second stage (Nawaz et al. 2023). Napier grass has been another focus, showing a biohythane production of 457 mL/g VS (Pomdaeng et al. 2022).

Furthermore, skim latex serum desulfated by rubber wood ash has demonstrated potential, with a biohythane yield of 367.56 mL/g COD, comprising 73.03 mL bioH_2_/g COD and 294.53 mL bioCH_4_/g COD (Raketh et al. 2022). Additionally, research on cassava starch-based polymers has revealed a biohythane production of 269 mL/g VS (Cremonez et al. 2020). Water hyacinth leaves, a type of invasive plant, have shown 195.1 mL/g VS of biohythane, including 51.7 mL bioH_2_/g VS and 143.4 mL bioCH_4_/g VS (Cheng et al. 2010). Lastly, investigations into spent mushroom beds have resulted in a biohythane production of 252.49 mL/g VS of biohythane (Bertasini et al. 2024). Table 7 provides a comprehensive overview of previous studies on two-stage bioH_2_ and bioCH_4_ (biohythane) production from various substrates.

**Table 7:** Studies on bioH_2_ and bioCH_4_ (biohythane) production from various substrates

Substrate	First stage			Second stage			Ref.
	Reactor/operation type	Operational conditions	BioH_2_ production	Reactor/operation type	Operational conditions	BioCH_4_ production	
Garbage slurry containing shredded office papers	CSTR	Temperature, 60 °C; pH, 5.8–6.0; HRT, 1.2 d; OLR, 97 kg COD/m^3^/d	5.4 m^3^/m^3^/d	IRPR	Temperature, 55 °C; HRT, 6.8 d; OLR, 15.7 kg COD/m^3^/d	6.1 m^3^/m^3^/d	Ueno et al. (2007a)
Cow dung + untreated domestic wastewater sludge	Batch	Temperature, 55 °C; pH, 5.5	108.04 mL/g VS	Batch	Temperature, 35 °C; pH, 7.5	768.54 mL/g VS	Sufyan et al. (2023)
Sedimented pulp and paper mill waste fiber	Batch	Temperature, 37 °C; initial pH, 6.4	42.1 mL/g VS	Batch	Temperature, 55 °C; initial pH, 7.1–8.0	334 mL/g VS	El-Qelish et al. (2024)
Tofu processing residue	Batch	S/I, 8 (VS basis); temperature, 37 °C; initial pH, 6	50 mL/g VS	Batch	S/I, 1 (VS basis); temperature, 37 °C; initial pH, 7	64.4 mL/g VS	Ali et al. (2024)
Organic solid waste	SBR	Temperature, 37 °C; initial pH, 5.5; HRT, 16 h; OLR, 60 g VS/L/d	28 mL/g VS	SBR	Temperature, 37 °C; initial pH, 7.5; HRT, 2.8 d; OLR, 2.8 g VS/L/d	230 mL/g VS	Salazar-Batres et al. (2024)
Residual glycerin from biodiesel	Batch	Temperature, 35 °C; initial pH, 6	247 mL/L_medium_	Batch	Temperature, 35 °C; initial pH, 7	729 mL/L_medium_	de Oliveira Faber et al. (2023)
Vinasse + spent brewer yeast	Batch	Temperature, room temperature; initial pH, 5.5	43.7 mL/g VS	Batch	Temperature, room temperature; initial pH, 7.5	214.6 mL/g VS	Nualsri et al. (2024)
High-strength industry food waste hydrolysate	Batch	Temperature, 37 °C; initial pH, 5.5	0.65 mL/g COD	Batch	Temperature, 37 °C; initial pH, 7–8	203.72 mL/g COD	Kongthong et al. (2023)
Food waste	Batch	Temperature, 37 °C; initial pH, 5.5	107.3 mL/g VS	Batch	Temperature, 37 °C; initial pH, 7	308 mL/g VS	Liu et al. (2022)
OFMSW	Batch	Temperature, 55 °C; fly ash addition, 19.2 g/L	95 mL/g VS	Batch	Temperature, 37 °C	400 mL/g VS	Alavi-Borazjani et al. (2023)
Food waste	LBR	Temperature, 37 °C; SRT, 6 d; OLR, 11.9 kg VS/m^3^/d	0.31 m^3^/kg VS	UASB	Temperature, 37 °C; HRT, 0.6 d; OLR, 5.4 kg VS/m^3^/d	0.21 m^3^/kg VS	Han and Shin (2004)
Wheat straw	Semi-continuous	Temperature, 35–37 °C; pH, 4.9–5.5	135 cm^3^	Semi-continuous	Temperature, 35–37 °C; pH, 5.5–7	95,168 cm^3^	Kabaivanova et al. (2022)
Corn steep liquor	Bioreactors with daily feeding mode	Temperature, 35 °C; pH, 5.5	670 cm^3^/L/d	Bioreactors with daily feeding mode	Temperature, 35 °C	220 cm^3^/L/d	Stoyancheva et al. (2023)
Wheat straw + horse manure	Batch	Temperature, 55 °C; initial pH, 5.5	0.29 cm^3^/g	Batch	Temperature, 55 °C; initial pH, 7.5	4.20 cm^3^/g	Hubenov et al. (2024)
Kitchen waste + municipal sewage sludge	Bioreactors with daily feeding mode	Temperature, 55 °C; initial pH, 10; OLR, 12.5 g VS/L/d	0.20 L/g VS	Bioreactors with daily feeding mode	Temperature, 55 °C; OLR, 5 g VS/L/d	0.73 L/g VS	Li et al. (2023)
Food waste	Batch	Temperature, 35 °C; initial pH, 7.5; S/I, 10 (VS basis)	102.79 mL/g VS	Batch	Temperature, 35 °C; initial pH, 7.5; tween 80 addition, 0.1% v/v	351.97 mL/g VS	Chen et al. (2023)
Glucose	Batch	Temperature, 35 °C; initial pH, 7; microalgae (*Chlorella* sp.) biochar addition, 10 g/L	117.17 mL/g glucose	Batch	Temperature, 35 °C; initial pH, 8	22.77 mL/g glucose	Guo et al. (2024)
Sugarcane leaves	Batch	Temperature, 37 °C; initial pH, 6.5	3187 mL/L	Batch	Temperature, 37 °C; initial pH, 7	5923 mL/L	Miftah et al. (2022)
Sorghum + cow manure	CSTR	Temperature, 37 °C; HRT, 5 d; OLR, 7.70 kg VS/m^3^/d (13.82 kg COD/m^3^/d)	209.2 mL/g carbohydrates_consumed_	CSTR	Temperature, 37 °C; HRT, 25 d; OLR, 1.49 kg VS/m^3^/d (1.78 kg COD/m^3^/d)	295.3 mL/g VS	Dareioti et al. (2022)
OFMSW	Batch	Temperature, 35 °C; S/I, 20 (VS basis)	41.7 mL/g VS	Biofilm reactor	Temperature, 35 °C; HRT, 1 d; OLR, 9 kg COD/m^3^/d	301 mL/g VS	Yeshanew et al. (2018)
Halophytic biomass (*Atriplexcrassifolia*)	Batch	Temperature, 37 °C; pH, 5.5	13.2 mL/g	Batch	Temperature, 45 °C; pH, 8	8.5 mL/mL	Nawaz et al. (2023)
Palm oil mill effluent	UASB	Temperature, 55 °C; initial pH, 5.5; HRT, 2 d; OLR, 75 g COD/L/d	215 L/kg COD	CSTR	Temperature, 37 °C; initial pH, 7.5; HRT, 5 d	320 L/kg COD	Krishnan et al. (2014)
Sugarcane leaf	Semi-CSTR	Temperature, 37 °C; initial pH, 7.2; HRT, 5 d; feeding rate, 0.20 L/d	60.1 mL/L/d	Semi-CSTR	Temperature, 37 °C; initial pH, 7.5; HRT, 25 d; feeding rate, 0.40 L/d	238.6 mL/L/d	Sukphun et al. (2023a)
Tomato plant residues	Batch	Temperature, 37 °C; initial pH, 6.5	11.6 mL/g VS	Batch	Temperature, 37 °C; S/I, 0.5 (VS basis)	252.3 mL/g VS	Ruiz-Aguilar et al. (2022)
Cheese whey	Biofilters	Temperature, 37 °C; HRT, 0.42 d	36.86 mL/g COD	Biofilters	Temperature, 55 °C; HRT, 2 d; OLR, 6.08 g COD/L/d	325 mL/g COD	Kovalev et al. (2022)
Second cheese whey	CSTR	HRT, 1 d; OLR, 10 g VS/L_/_d	0.12 L/g VS	CSTR	HRT, 7.5 d; OLR, 0.67 g VS/L/d	0.34 L/g VS	Lembo et al. (2022)
Sucrose	CSTR	Temperature, 35 °C; pH, 5.2–5.3; HRT, 12 h; OLR, 22.5 g COD/L/d	189 mL/g COD	Upflow anaerobic filter	Temperature, room temperature; pH, 6.5–8; HRT, 2 d; OLR, 4.3 g COD/L/d	323 mL/g COD	Kyazze et al. (2007)
Cheese whey	CSTR	Temperature, 35 °C; HRT, 24 h	2.51 L/d; 0.041 m^3^/kg COD	PABR	Temperature, 35 °C; HRT, 4.4 d	75.6 L/d	Moreno-Andrade et al. (2015)
OFMSW (rice portion)	Batch	Temperature, 37 °C; initial pH, 5.5	125 mL/g VS	Batch	Temperature, 37 °C; initial pH, 6.5	232 mL/g VS	Dong et al. (2011)
Cheese whey	AnSTBR	Temperature, 25 °C; HRT, 12 h; OLR, 50 kg COD/m^3^/d	1.11 mol/mol lactose	AnSTBR	Temperature, 25 °C; HRT, 43 h; OLR, 5 kg COD/m^3^/d	170 mL/g/COD	Blanco et al. (2023)
Catering waste	Batch	Temperature, 37 °C; initial pH, 6.5	5.9 mL/g VS	Batch	Temperature, 37 °C; initial pH, 7.5	17.7 mL/g VS	Elwakeel et al. (2023)
Catering waste	CSTR	Temperature, 37 °C; Initial pH, 6.5; HRT, 1 d; OLR, 91.5 g COD/L/d	22.24 mL/g VS	CSTR	Temperature, 37 °C; HRT, 10 d; OLR, 10 g COD/L/d	98.7 mL/g VS	Elwakeel et al. (2023)
Food waste + chicken manure + Corn straw	CSTR	Temperature, 55 °C; HRT, 4 d; OLR, 8 g VS/L/d	0.699 L/L/d	Bubble reactor	Temperature, 55 °C; HRT, 12 d	0.690 L/L/d	Yellezuome et al. (2024)
OFMSW	Semi-CSTR	Temperature, 55 °C; HRT, 2 d; OLR, 3 g VS/L/d; rice husk ash addition, 5 g/L	184.7 mL/g VS	Semi-CSTR	Temperature, 37 °C; HRT, 12 d; OLR, 0.44 g VS/L/d	727.2 mL/g VS	Alavi-borazjani et al. (2022)
High-strength organic wastewater	Semi-continuous	Temperature, 37 °C; initial pH, 5.5; HRT, 10 d; OLR, 25 g COD/L/d	1.804 L/L/d; 1.641 mol/mol glucose	Semi-continuous	Temperature, 37 °C; HRT, 15 d; OLR, 15.7 g COD/L/d	1.003 L/L/d	Wirasembada et al. (2024)
Napier grass	Semi-CSTR	Temperature, 38 °C; OLR, 1 kg VS/m^3^/d; pH, 5.5	90 mL/g VS	Semi-CSTR	Temperature, 38 °C; OLR, 0.5 kg VS/m^3^/d	367 mL/g VS	Pomdaeng et al. (2022)
Food waste	Batch	Temperature, 36 °C; operational pH, 5.7	23.2 mL/g VS	Batch	Temperature, 36 °C; SIR, 0.25 (VS basis)	408.7 mL/g VS	García-Depraect et al. (2022)
Tofu processing residue	Batch	Temperature, 37 °C; initial pH, 7; operational pH, 5–6; S/I, 8 (VS basis); FeCl_3_ addition, 0.6 g/L	59.82 mL/g VS	Batch	Temperature, 37 °C; operational pH, 7; S/I, 0.5 (VS basis)	521.60 mL/g VS	Ali et al. (2022)
*Agave tequilana* bagasse	CSTR	Temperature, 35 °C; HRT, 6 h; OLR, 44 g COD/L/d	6 L/L/d	UASB	Temperature, room temperature (23–25 °C); HRT, 14 h; OLR, 20 g COD/L/d	6.4 L/L/d	Montiel Corona and Razo-Flores (2018)
Macro-algae (*Laminaria digitata*) + micro-algae (*Nannochloropsis oceanica*)	Batch	Temperature, 37 °C; initial pH, 6;	94.5 mL/g VS	Batch	Temperature, 37 °C; initial pH, 8; S/I, 0.5 (VS basis)	295.9 mL/g VS	Ding et al. (2016)
Stillage	CSTR	Temperature, 55 °C; initial pH, 6; HRT, 3 d; feed rate, 400 mL/d	69 mL/g VS	CSTR	Temperature, 55 °C; HRT, 12 d; feed rate, 292 mL/d	348 mL/g VS	Luo et al. (2011)
Glucose	Batch	Temperature, 35 °C; initial pH, 7	185 L/kg COD	Batch	Temperature, 35 °C; initial pH, 7	267 L/kg COD	Giordano et al. (2011)
Food industry waste (durum wheat)	Batch	Temperature, 35 °C; Initial pH, 7	76 L/kg COD	Batch	Temperature, 35 °C; initial pH, 7	243 L/kg COD	Giordano et al. (2011)
Cassava stillage + excess sludge	Batch	Temperature, 60 °C; initial pH, 6	31.3 mL/g VS	Batch	Temperature, 60 °C ; initial pH, 7.5	195.5 mL/g VS	Wang et al. (2011)
Cassava stillage + excess sludge	Semi-CSTR	Temperature, 60 °C; HRT, 3 d	74.0 mL/g VS_added_	Semi-CSTR	Temperature, 60 °C; HRT, 12.5 d	338.9 mL/g VS	Wang et al. (2011)
Wheat straw	UASB	Temperature, 70 °C; HRT, 1 d; OLR, 9.9 g VS/L/d	89 mL/g VS	UASB	Temperature, 55 °C; HRT, 3 d; OLR, 3.3 g VS/L/d	307 mL/g VS	Kongjan et al. (2011)
Food waste	Semi-continuous reactor	Temperature, 55 °C; HRT, 1.9 d; OLR, 39 g COD/L/d	11.1 L/L_fed_/d	Biogas sparging-type reactor	Temperature, 55 °C; HRT, 15.4 d; OLR, 4.16 g COD/L/d	47.4 L/L_fed_/d	Lee et al. (2010)
Olive pulp	CSTR	Temperature, 35 °C; HRT, 14.5 h; feed rate, 46.3 g TS/d	0.13 mole/kg TS	CSTR	Temperature, 35 °C; HRT, 20 d; SRT, 23 d; OLR, 3.95 g COD/L/d	0.16 L/kg COD	Koutrouli et al. (2009)
Cheese whey	CSTR	Temperature, 35 °C; HRT, 24 h; pH, 5.2	0.78 mol/mol glucose_consumed_	CSTR	Temperature, 35 °C; HRT, 20 d; pH, 4.9	147 mL/g VSS	Venetsaneas et al. (2009)
Potatoes	Batch	Temperature, 35 °; operational pH, 6	271.2 mL/g VS	Batch	Temperature, 35 °C; initial pH, 8	157.9 mL/g VS	Xie et al. (2008a)
Sweet sorghum	CSTR	Temperature, 35 °C; pH, 4.7–5.5; HRT, 12 h	10.4 L/kg sweet sorghum	CSTR	Temperature, 35 °C; influent pH, 4.7; HRT, 20 d	29 L/kg sweet sorghum	Antonopoulou et al. (2008)
Artificial garbage slurry containing milled paper	Jar fermenter	Temperature, 60 °C; pH, 6; HRT, 12 h; OLR, 74.3 g/L/d	199 mmol/L_reactor/_d	TDAPR	Temperature, 55 °C; pH, not adjusted; HRT, 13 h; OLR, 76.9 g/L/d	442 mmol/L_reactor/_d	Ueno et al. (2007b)
Disposable nappies + fruit/vegetable waste	CSTR	Temperature, 37 °C; HRT, 2 d; OLR, 16.75 kg COD/m^3^/d; recirculation ratio, 0.5	0.88 L/L_fed_	CSTR	Temperature, 37 °C; HRT, 25 d; OLR, 1.32 kg COD/m^3^/d	0.31 L/g COD_removed_	Tsigkou et al. (2023)
Food waste	CSTR	Temperature, 35 °C; pH, 5.5; HRT, 3 d; OLR, 14.2 kg TVS/m^3^/d	12.6 L/kg TVS/d	CSTR	Temperature, 35 °C; HRT, 12.8 d; OLR, 2.5 kg TVS/m^3^/d	482.1 L/kg TVS/d	Baldi et al. (2019)
Food waste + activated sludge	CSTR	Temperature, 35 °C; pH, 5.5; HRT, 3 d; OLR, 14.6 kg TVS/m^3^/d	8.6 L/kg TVS/d	CSTR	Temperature, 35 °C; HRT, 11.9 d; OLR, 2.5 kg TVS/m^3^/d	428.3 L/kg TVS/d	Baldi et al. (2019)
Food waste	CSTR	Temperature, 55 °C; HRT, 5 d; OLR, 18 kg VS/m^3^/d; recirculation ratio, 0.3	135 L/kg/VS	CSTR	Temperature, 35 °C; HRT, 9 d; OLR, 5.7 kg VS/m^3^/d	510 L/kg/VS	Algapani et al. (2019)
Microalgae + fecal sludge	Multi-batch reactor system	Temperature, 30 °C; initial pH, 6	24.5 mL/g VS	Multi-batch reactor system	Temperature, 30 °C; initial pH, 7.5	281 mL/g VS	Khalekuzzaman et al. (2023)
Grass silage + cattle slurry	Batch	Temperature, 55 °C; Initial pH, 6; biochar addition, 10 g/L	29.6 L/kg VS	Batch	Temperature, 37 °C; initial pH, 7; SIR, 0.5 (VS basis)	253 L/kg VS	Ning et al. (2023b)
Grass silage + Cattle slurry	CSTR	Temperature, 55 °C; pH, 5.5; HRT, 4 d; OLR, 14 g VS/L/d; biochar addition, 10 g/L	11.5 L/kg VS	CSTR	Temperature, 37 °C; HRT, 16; OLR, 4 g VS/L/d; biochar addition, 10 g/L	236.9 L/kg VS	Ning et al. (2023a)
Date fruit wastes	Semi-CSTR	Temperature, 37 °C; pH, 6; HRT, 118 h; OLR, 11.74 g VS/L/d	0.081 L/g VS	Semi-CSTR	Temperature, 37 °C; pH, 7.3; HRT, 8 d; OLR, 4.25 g VS/L/d	0.530 L/g VS	Saidi et al. (2023)
Skim latex serum desulfated by 10 g/L rubber wood ash	Batch	Temperature, 55 °C; initial pH, 7; S/I, 70/30% v/v	73.03 mL/g COD	Batch	Temperature, 35 °C; initial pH, 7.55; S/I, 30/70% v/v	294.53 mL/g COD	Raketh et al. (2022)
Cassava starch-based polymer	Semi-continuous	Temperature, 37 °C; HRT, 5 d; pH, 5–6	19.9 mL/g VS	Semi-continuous	Temperature, 37 °C; HRT, 20 d; pH, 7–8	249.1 mL/g VS	Cremonez et al. (2020)
OFMSW	Batch	Temperature, 55 °C; S/I, 2 (VS basis); nano-sized fly ash addition, 5 g/L	123 mL/g VS	Batch	Temperature, 37 °C; S/I, 4 (VS basis)	676 mL/g VS	Alavi-Borazjani et al. (2024)
Sugarcane straw hemicellulose hydrolyzate	Batch	Temperature, 35 °C; Initial pH, 5.5; initial carbohydrates concentration, 9 mmol/L	177.16 mL/g COD	Batch	Temperature, 35 °C; initial pH, 7	110.59 mL/g COD	Tomasini et al. (2023)
Swine manure	CSTR	Temperature, 37 °C; initial pH, 5.5; HRT, 3 h	1.8 mL/L/d	CSTR	Temperature, 55 °C; initial pH, 7; HRT, 10.95 d	65.7 mL/L/d	Chu et al. (2022)
Sugarcane bagasse	Batch	Temperature, 37 °C; initial pH, 6;	396.50 mL/L/d	Batch	Temperature, 37 °C; initial pH, 7.59; Ni concentrations, 7.59 mg/L; Fe concentrations, 3.61 mg/L	185.73 mL/L/d	Thungklin et al. (2018)
Food waste + Sewage sludge	Semi-continuous	Temperature, 37 °C; pH_feed_, 4.6; HRT, 5 d; OLR, 5.9 g VS/L/d; C/N, 20	129.1 mL/g VS_removed_	Semi-continuous	Temperature, 37 °C; pH_feed_, 5.3; HRT, 10 d; OLR, 1.59 g VS/L/d; C/N, 18	617.6 mL/g VS_removed_	Siddiqui et al. (2011)
Cassava stillage	CSTR	Temperature, 60 °C; HRT, 1 d	56.6 mL/g VS	CSTR	Temperature, 55 °C; HRT, 5 d	249 mL/g VS	Luo et al. (2010)
Glucose synthetic medium	Batch	Temperature, 35 °C; initial pH, 7.2	442 mmol/L	Batch	Temperature, 35 °C; initial pH, 7.2	530 mmol/L	Ruggeri et al. (2010)
Protein-mixed food waste	Batch	Temperature, 37 °C; operational pH, 6	186.1 ml/g TVS	Batch	Temperature, 37 °C; initial pH, 8	209.7 ml/g TVS	Song et al. (2010)
Synthetic landfill leachate	CSTR	Temperature, 37 °C; HRT, 8 h; SRT, 2.2 d; OLR, 22.5 g COD/L/d; pH, 5.5–6.5	400 mL/g glucose	CSTR	Temperature, 37 °C; HRT, 10 d; SRT, 10 d; OLR, 0.6 g COD/L/d; pH, 6.8–7.2	426 mL/g COD_removed_	Hafez et al. (2010)
Water hyacinth leaves	Batch	Temperature, 35 °C; initial pH, 6	51.7 mL/g TVS	Batch	Temperature, 35 °C; initial pH, 8	143.4 mL/g TVS	Cheng et al. (2010)
Food waste	SCRD	Temperature, 40 °C; SRT, 160 h; OLR, 22.65 kg VS/m^3^/d	0.065 m^3^/kg VS	Semi-CSTR	Temperature, 40 °C; SRT, 26.67 d; OLR, 4.61 kg VS/m^3^/d	0.546 m^3^/kg VS	Wang and Zhao (2009)
Glucose	Batch	Temperature, 35 °C; operational pH, 6; S/I, 0.5 (mass basis)	2.75 mol/mol glucose	Batch	Temperature, 35 °C; initial pH, 8; S/I, 1 (mass basis)	2.13 mol/mol glucose	Xie et al. (2008b)
Food waste	Continuous	Temperature, 55 °C; pH, 5.5; HRT, 1.3 d; OLR, 38.4 kg VS/m^3^/d	205 mL/g VS	Continuous	Temperature, 35 °C; HRT, 5 d; OLR, 6.6 kg VS/m^3^/d	464 mL/g VS	Chu et al. (2008)
Food waste	Batch	Temperature, 35 °C; S/I, 0.2 v/v; ZVI addition, 10 g/L; NaCl addition, 9 g/L	303.38 mL/g VS	Batch	Temperature, 35 °C; initial pH, 7	253.84 mL/g VS	Chen et al. (2024)
Cheese whey + Glycerin	AFBR	Temperature, 55 °C; HRT, 4 h; OLR, 11.6 kg COD/m^3/^d	9.9 mol/kg COD_removed_	AFBR	Temperature, 30 °C; HRT, 24 h; OLR, 18.5 kg COD/m^3/^d	14 mol/kg COD_removed_	Almeida et al. (2023)
Palm oil mill effluent + Concentrated latex wastewater	CSTR	Temperature, 55 °C; HRT, 7 d; OLR, 3.14 g VS/L/d	95.45 mL/g VS	UASB	Temperature, 30 °C; HRT, 15 d; OLR, 3.14 g VS/L/d	1.20 mL/g VS	Raketh et al. (2023)
Cheese whey	AnSBBR	Temperature, 55 °C; feeding rate, 661 kg COD/d	11.1 kmol/d	AnSBBR	Temperature, 55°C; feeding rate, 89.8 kg COD/d	0.885 kmol/d	Batista et al. (2023)
Food waste	Batch	Temperature, 37 °C; initial pH, 6.5	154.91 mL/g VS	Batch	Temperature, 37 °C; initial pH, 7.5	381.83 mL/g VS	Gu et al. (2024)
Food waste	Continuous	Temperature, 35 °C; HRT, 3 d	38.1 mL/g VS	Continuous	Temperature, 35 °C; HRT, 15 d	439.6 mL/g VS	Ding et al. (2022)
Sugarcane bagasse	Batch	Temperature, 37 °C; initial pH, 6.8	166.8 mL/L	Batch	Temperature, 37 °C	870.8 mL/L	Silva Rabelo et al. (2023)
Municipal food waste + Sewage sludge	Semi-continuous	Temperature, 35 °C; initial pH, 7; SRT, 1.25 d	0.93 mL/mL feedstock	Semi-continuous	Temperature, 35 °C; SRT, 12.5 d	9.5 mL/mL feedstock	Zhu et al. (2011)
Municipal biowaste	CSTR	Temperature, 55 °C; HRT, 3.3 d; OLR, 16 kg VS/m^3^/d	51 L/kg VS	CSTR	Temperature, 55 °C; HRT, 12.6 d; OLR, 4 kg VS/m^3^/d	416 L/kg VS	Cavinato et al. (2011)
Potato waste	CSTR	Temperature, 35 °C; pH, 5.5; HRT, 6 h	30 L/kg TS	CSTR	Temperature, 35 °C; pH, 7; HRT, 90 h	183 L/kg TS	Zhu et al. (2008)
Microalgae (*Chlorella* sp.)	Batch	Temperature, 37 °C; initial pH, 6	9 mL/g VS	Batch	Temperature, 37 °C; initial pH, 7; S/I, 0.5 (VS basis)	311.9 mL/g VS	Wu et al. (2023)
Synthetic wastewater	Suspended-growth reactor	Temperature, 35 °C; HRT=SRT, 1 d; feeding rate, 0.225 L/d	5.64 mL/g COD	Suspended-growth reactor	Temperature, 35 °C; HRT=SRT, 20 d; feeding rate, 0.2 L/d	320 mL/g COD	DiStefano and Palomar (2010)
Household solid waste	CSTR	Temperature, 37 °C; SRT, 2 d; OLR, 37.5 kg VS/m^3^/d	43 mL/g VS	CSTR	Temperature, 37 °C; SRT, 15 d; OLR, 4.1 kg VS/m^3^/d	500 mL/g VS	Liu et al. (2006)
Food waste hydrolysate	Batch	Temperature, 35 °C; pH, 5.5	21.33 mL/g VS	Batch	Temperature, 35 °C; pH, 7.2	373.83 mL/g VS	Pomdaeng et al. (2024)
Synthetic wastewater	AnSBBR	Temperature, 28 °C; operational pH, 6.0; OLR, 4.75 kg COD/m^3^/d	1.59 mol/kg COD_removed_	AnSBBR	Temperature, 28 °C; operational pH, 7.0; OLR, 1.81 kg COD/m^3^/d	0.363 m^3^/kg COD_removed_	Venkata Mohan et al. (2008)
Food waste	Batch	Temperature, 35 °C; initial pH, 6	73.64 mL/g VS	Batch	Temperature, 35 °C; initial pH, 8	139.24 mL/g VS	Cheng et al. (2016)
Sewage sludge	Batch	Temperature, 35 °C; initial pH, 6	38.80 mL/g VS	Batch	Temperature, 35 °C; initial pH, 8	96.90 mL/g VS	Cheng et al. (2016)
Sewage sludge + Wine vinasse + Poultry manure	CSTR	Temperature, 55 °C; HRT, 5 d; S/I, 4 (mass basis)	40.41 mL/g VS	CSTR	Temperature, 35 °C; HRT, 12 d; S/I, 4 (mass basis)	391.0 mL/g VS	Sillero et al. (2024)
Food waste + Chicken manure + Corn straw	Batch	Temperature, 55 °C; initial pH, 5.5; S/I,2 (VS basis)	81.54 mL/g VS	Batch	Temperature, 55 °C; initial pH, 7.5; S/I,1 (VS basis)	550.98 mL/g VS	Liu et al. (2023)
Spent mushroom bed	Semi-continuous	Temperature, 38 °C; pH, 5.5–6.5; HRT, 6 d; OLR, 7.2 g COD/L/d	47.88 L/Kg VS	Batch	Temperature, 38 °C; S/I, 1 (VS basis)	204.61 L/Kg VS	Bertasini et al. (2024)
Microalgae (*S.obliquus*)	Batch	Temperature, 37 °C	56 cm^3^/g VS	Batch	Temperature, 37 °C	119 cm^3^/g VS	Abimbola et al. (2023)
Pre-aerated protein-rich food waste	Batch	Temperature, 35 °C; initial pH, 6; S/I, 0.3 (VS basis)	5.27 ml/g VS	Batch	Temperature, 35 °C; S/I, 0.3 (VS basis)	351.69 ml/g VS	Rafieenia et al. (2017)

*S/I* substrate-to-inoculum ratio, *IRPR* internal recirculation packed-bed reactor, *LBR* leaching-bed reactor, *SBR* sequencing batch reactor, *IABR* integrated anaerobic bioreactor, *PABR* periodic anaerobic baffled reactor, *AnSTBR* anaerobic structured-bed reactor, *TDAPR* thermophilic down-flow anaerobic packed-bed reactor, *SCRD* semi-continuous rotating drum, *AFBR* anaerobic fluidized bed reactor, *AnSBBR* anaerobic sequencing batch biofilm reactor, *ZVI* zero-valent iron

## Bioreactor design/configurations

Recent research provides strong evidence in favor of utilizing a sequential two-stage AD process instead of a single-stage approach for maximizing biohythane production (Sasidhar et al. 2022). Therefore, to achieve the highest bioH_2_ and bioCH_4_ yields in the two-stage process and to enable large-scale industrial biohythane production, the effective integration of bioreactors is crucial.

**Fig. 6:**
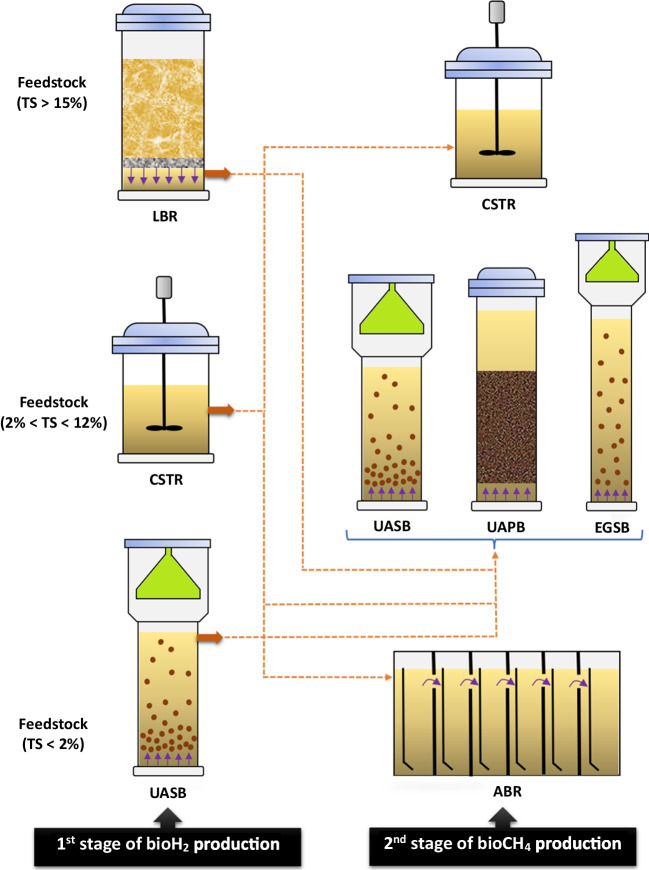
Proposed reactor integrations for sequential bioH_2_ and bioCH_4_ generation based on the feedstock concentration (adapted and redrawn from Liu et al. (2013)). ABR anaerobic baffled reactor, CSTR continuously stirred tank reactor, EGSB expanded granular sludge bed reactor, LBR leaching-bed reactor, UAPB up-flow anaerobic packed-bed reactor, UASB up-flow anaerobic sludge blanket reactor

The selection of bioreactor configuration in the two-stage biohythane production process is influenced by various factors, especially the properties of the feedstock utilized (Rajendran et al. 2020). Figure 6 depicts the potential reactor integration strategies proposed for two-stage biohythane production, which are dependent on the feedstock concentration. For feedstocks with a high total solid (TS) content exceeding 15% (dry basis), a Leaching Bed Reactor (LBR) is a suitable choice for the initial biohydrogen production stage. The resulting leachate can then be processed in high-performance anaerobic reactors such as the Up-flow Anaerobic Sludge Blanket (UASB), Up-flow anaerobic packed-bed (UAPB), and expanded granular sludge bed (EGSB) for bioCH_4_ generation in the subsequent stage (Liu et al. 2013). When the feedstock’s TS falls within the moderate range of 2–12%, continuous stirred-tank reactors (CSTRs) are generally considered the preferred option for bioH_2_ production. For the next methanogenic stage in this case, different reactor configurations can be chosen depending on the characteristics of the digestate produced in the initial stage. Regarding substrates with a very low TS content (less than 2%), high-rate anaerobic reactors like UASB can be employed for both biohydrogenation and biomethanation stages (Abdur Rawoof et al. 2021).

In practice, different combinations of reactors have been used in two-stage AD systems. While batch mode may be easier to operate and more economically feasible, continuous mode has been shown to be more efficient (Meena et al. 2020). Among bioreactor types, CSTR stands out as the most commonly employed for both bioH_2_ and bioCH_4_ production stages (Mozhiarasi et al. 2023) due to its advantages such as ease of setup and capacity to facilitate direct contact between the substrate and active biomass through complete mixing (Nageswara-Rao and Soneji 2018). For example, Cavinato et al. (2011) demonstrated the effectiveness of a two-stage CSTR system operating at thermophilic conditions (55 °C) using municipal biowaste as a substrate. The first stage, prioritizing bioH_2_ production, employed a shorter HRT (3.3 days) and a higher OLR (16 kg VS/m^3^/d), achieving a bioH_2_ yield of 51 L/kg VS. In contrast, the second stage focused on maximizing bioCH_4_ yield by using a longer HRT (12.6 days) and a lower OLR (4 kg VS/m^3^/d), resulting in a significant increase in bioCH_4_ production to 416 L/kg VS. Similarly, potato waste was processed using a two-stage system with CSTRs at mesophilic conditions (35 °C). The initial stage, with an initial pH of 5.5 and an HRT of 6 h, yielded 30 L bioH_2_/kg TS, while the second stage controlled at a neutral pH of 7 and an HRT of 90 days produced 183 L bioCH_4_/kg TS (Zhu et al. 2008). For a combination of wine vinasse, sewage sludge, and poultry manure, an integrated two-stage system using CSTRs showed that operating the first stage at 55 °C with an HRT of 5 days achieved 40.41 mL bioH_2_/g VS. The second stage operated at 35 °C with an HRT of 12 days, yielded 391 mL bioCH_4_/g VS. Additionally, a combination of CSTR and UASB reactor was employed in some studies. For instance, in a UASB-CSTR system treating palm oil mill effluent, the first UASB stage operated at 55 °C and an HRT of 2 days, yielding 215 L bioH_2_/kg COD, while the subsequent CSTR stage at 37 °C with an HRT of 5 days generated 320 L bioCH_4_/kg COD (Krishnan et al. 2014). A two-stage approach utilizing a CSTR followed by a UASB reactor was also explored for *Agave tequilana* bagasse (Montiel Corona and Razo-Flores 2018). In the initial CSTR stage maintained at 35 °C, a shorter HRT of 6 h and a higher OLR of 44 g COD/L/d facilitated bioH_2_ production, resulting in a bioH_2_ yield of about 6 L/L/d. Subsequently, the process transitioned to a UASB reactor operating at a lower temperature range (23–25 °C) with a longer HRT of 14 h and a reduced OLR of 20 g COD/L/d, achieving a bioCH_4_ yield of approximately 6.4 L/L/d.

Apart from the aforementioned systems, numerous other reactor configurations have proven effective in two-stage AD processes. For example, Salazar-Batres et al. (2024) investigated a two-stage SBR-SBR (Sequencing Batch Reactor) system for treating organic solid waste. Both stages were maintained at a constant temperature of 37 °C. The first stage operated with a 16-h HRT and a high OLR of 60 g VS/L/d, promoting bioH_2_ production at 28 mL/g VS. The subsequent stage utilized a longer HRT of 2.8 days and a lower OLR of 2.8 g VS/L/d, focusing on bioCH_4_ generation with a yield of 230 mL/g VS. Additionally, synthetic wastewater was processed using an SBBR-SBBR (Sequencing Batch Biofilm Reactor) configuration. The first SBBR operated at 28 °C with a higher OLR of 4.75 kg COD/m^3^/d compared to the second bioreactor, resulting in the production of 1.59 moles of bioH_₂_/kg COD_removed_. The second stage, also operating at 28 °C but with an OLR of 1.81 kg COD/m^3^/d, yielded 0.363 m^3^ of bioCH_4_/kg COD_removed_ (Venkata Mohan et al. 2008). In another study, synthetic wastewater was treated using a two-stage mesophilic system with suspended-growth reactors in both stages. In the first stage, HRT and SRT were both set to 1 day, with a feed rate of 0.225 L/d. This stage resulted in a bioH_2_ production of 5.64 mL/g COD. The second stage, with an HRT and SRT of 20 days and a feed rate of 0.2 L/d, produced 320 mL of bioCH_4_/g COD (DiStefano and Palomar 2010). Furthermore, wheat straw underwent treatment in a dual-stage UASB configuration. The initial phase functioned at 70 °C with a 1-day HRT and 9.9 g VS/L/d OLR, achieving a bioH_2_ production of 89 mL/g VS. The second stage UASB functioned at 55 °C with a 3-day HRT and 3.3 g VS/L/d OLR, resulting in a bioCH_4_ yield of 307 mL/g VS (Kongjan et al. 2011). Additionally, de Souza Almeida et al. (2023) investigated a two-stage AFBR (Anaerobic Fluidized Bed Reactor) system using a cheese whey-glycerin mixture. The initial phase was conducted at 55 °C with a 4-h HRT and 11.6 kg COD/m^3^/d OLR, resulting in 9.9 moles of bioH_2_/kg COD_removed_. The second phase, operating at 30 °C with a 24-h HRT and an 18.5 kg COD/m^3^/d OLR, produced 14 moles of bioCH_4_/kg COD_removed_. Table 8 outlines the main advantages and disadvantages of bioreactors utilized in previous studies for biohythane production.

**Table 8:** Advantages and disadvantages of various bioreactors employed in biohythane production

Reactor type	Advantages	Disadvantages	Ref.
CSTR	- Ease of setup and operation - Uniform mixing - Enhancing mass transfer and direct contact between substrate and active biomass - Effective for washing out methanogens in the first hydrogenogenic stage	- Long retention time and large reactor volume - Energy intensive - Potential disruption of microbial flocs at high rotational speeds - Risk of biomass washout at short HRTs - Risk of short-circuit currents and flushing out undigested substrate without proper stirring	Skiadas et al. (2003); Show et al. (2011); Nageswara-Rao and Soneji (2018); Hans and Kumar (2019); Alavi-Borazjani et al. (2020); Martinez-Burgos et al. (2021); Dangol et al. (2022); Holl et al. (2022); Singh et al. (2023)
UASB	- Equipment versatility - Low operational cost - Suitable for feedstocks with low TS content - Retaining high biomass concentration - Handling high OLRs - Low sludge production	- Long start-up time - No guarantee of success for the self-immobilization process - Vulnerability to sudden shock loads - Filtration requirement for large particles when treating certain substrates - Requirement for effluent post-treatment	Veronez et al. (2005); Tiwari et al. (2006); Show et al. (2011); Abdur Rawoof et al. (2021); Martinez-Burgos et al. (2021); Holl et al. (2022)
LBR	- Suitable for feedstocks with high TS content - Low energy and water demand - Effective in separating dissolved organic compounds - Preventing the loss of partially decomposed substrate and active biomass - Leachate recycling without utilizing the solid–liquid separation unit	- Inefficient hydrolysis of complex feedstocks - Sophisticated operational condition control - Diminished leachate permeability or bed clogging at elevated OLRs	Liu et al. (2013); Dangol et al. (2022); Holl et al. (2022); Singh et al. (2023)
ASBR	- Simple design - No need to external filtration - Providing long retention times for solids - No short circuit - Biomass retention without using fixed media or a solid settling chamber - No need to primary and secondary settles - Possibility of intermittent operation - Efficient operating control - Highly stable under hydraulic shock loads	- Pressure variations during feeding and withdrawal - Precise agitation requirements - Challenging to decant - Risk of reactor structure damage due to negative pressure	Sarti et al. (2007); Pinheiro et al. (2008); Shao et al. (2008); Yang et al. (2019); Holl et al. (2022)
AFBR	- Appropriate for substrates with low organic content - Providing a large surface area for attached biological growth - Allowing high biomass concentration - Lower reactor volume - No need for mechanical mixing - Enabling the application of high OLRs and short HRTs - High flow velocity - Maintaining stability under varying feed conditions or toxic shocks - Reduced sludge production - No problem of biomass washout - Overcoming operating problems like bed clogging and high pressure drop	- Limited suitability for treating substrates with high suspended solids - Costliness of the carrier medium and support system - Elevated power demands for bed expansion or fluidization - Bioparticle washout risk with small, high-density supports - Support material size reduction due to system turbulence	Heijnen et al. (1989); Skiadas et al. (2003); Saravanan and Sreekrishnan (2006); Barros et al. (2010); Carbajo et al. (2010); Andalib et al. (2012); Jaafari et al. (2014); Chaves et al. (2021); Martinez-Burgos et al. (2021)

*CSTR* continuous stirred-tank reactor, *UASB* upflow anaerobic sludge blanket, *LBR* leach-bed reactor, *ASBR* anaerobic sequencing batch reactor, *AFBR* anaerobic fluidized bed reactor

## Main challenges for biohythane scale-up

**Fig. 7:**
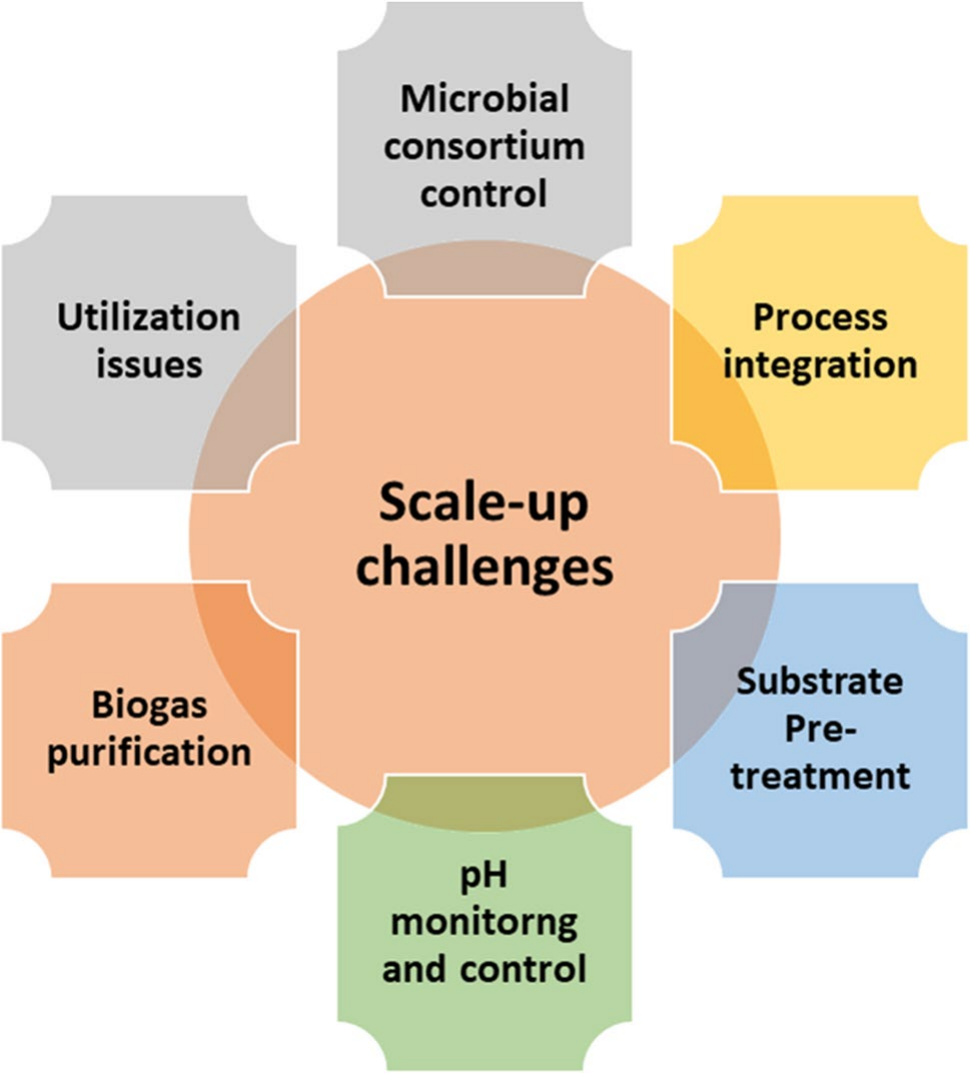
Main challenges for scaling up the biohythane production process

While there are numerous benefits to biohythane production using the AD process, it is important to acknowledge and address the various limitations associated with this technology. Overcoming these challenges is crucial for the successful implementation of biohythane production on a larger scale, allowing for the efficient recovery of biohythane from the substantial volumes of organic waste produced globally. Figure 7 illustrates the main challenges in scaling up the biohythane production process, with further explanations provided below.

BioH_2_ is an intermediate product of biomethanation, and to recover it as part of biohythane, the hydrogenotrophic methanogenesis pathway must be inhibited (Liu et al. 2018; Krishnan et al. 2019). A key challenge in achieving this lies in managing the microbial community, particularly during the initial hydrogenogenic phase (Mozhiarasi et al. 2023). However, using mixed microflora in the fermentation process may be inefficient due to bioH_2_ consumers like hydrogenotrophic methanogens, requiring pre-treatment measures and making the process economically unfeasible (Shanmugam et al. 2021a). Selecting an appropriate pre-treatment method poses technical challenges as not all bioH_2_ producers can form endospores. Conversely, some bioH_2_ consumers, like acetogens, can form spores, adding complexity to the process (Krishnan et al. 2019). While using pure cultures has been effective in enhancing bioH_2_ production, it is not practical for large-scale implementation (Krishnan et al. 2019). Therefore, using mixed microbial consortia is preferred over pure cultures to reduce costs. However, ensuring the availability of mixed consortia rich in bioH_2_-producing microorganisms during scale-up is essential.

The efficient integration of the hydrogenogenic and methanogenic stages is crucial for successful biohythane production. The functioning of the bioCH_4_-producing reactor relies on the bioH_2_-producing reactor, where the used medium from the initial phase acts as the methanogens’ substrate in the subsequent phase. Nonetheless, transferring feedstock continuously between the first- and second-stage reactors poses a challenge, requiring a sophisticated control system (Mozhiarasi et al. 2023). Moreover, additional energy inputs are required for tasks like heating, mixing, and pumping, and energy losses can occur due to phase changes and product release (Liu et al. 2018). Overall, the separate production of bioH_2_ and bioCH_4_ in distinct bioreactors is technically complex and involves high investment, operating, and maintenance costs (Abdur Rawoof et al. 2021). Therefore, integrating the two stages into a single digestion unit can help alleviate these challenges significantly (Shanmugam et al. 2021a).

Substrates with a resistant structure significantly reduce the hydrolysis rate, hindering biohythane production. Therefore, pre-treatment of these substrates is crucial to enhance their biodegradability. Researchers have proposed various approaches for this purpose, including physical, chemical, and biological methods. However, choosing the appropriate pre-treatment method for these challenging substrates is still a difficult task. This is because each method has its own strengths and weaknesses, making it difficult to find a universal approach that can effectively treat all types of feedstocks.

Accurate monitoring and control of pH are essential for efficient bioH_2_ production and subsequent bioCH_4_ generation. In laboratory settings, commercial chemicals are commonly used for pH control. While adding external chemicals for pH control may be straightforward in small-scale biohythane production setups, it becomes more difficult and costly in large-scale plants. Excessive alkali addition in such operations can result in a digestate with a high salt concentration, reducing its potential as a biofertilizer. Additionally, using chemicals for pH regulation can lead to high concentrations of metal ions and ammonia, presenting further challenges (Krishnan et al. 2019). Therefore, it is crucial to explore alternative approaches for pH control in large-scale operations to address these issues and ensure efficient and sustainable biohythane production.

It is essential to purify biogas from both the hydrogenogenic and methanogenic stages to ensure that only pure bioH_2_ and bioCH_4_ gases are suitable for use in the biohythane blend. Several techniques have been developed for biogas purification over the years, with some currently in use on an industrial scale. However, the economic viability of biohythane production is hindered by the high energy and chemical requirements of existing purification methods. Therefore, it is essential to prioritize the development of sustainable and energy-efficient approaches for biohythane purification to address these challenges and establish a more economically feasible pathway for biohythane production.

In addition to the points mentioned above, there are significant challenges related to the use of biohythane and the handling of by-products from the AD process. A key obstacle is the current gas distribution system and the lack of developed filling stations, which hinder the widespread commercialization of biohythane. The absence of enforced standards further complicates the situation, highlighting the need for regulatory frameworks and guidelines to facilitate its market integration (Mozhiarasi et al. 2023). Managing digestate, the primary by-product of biohythane production, is also a major challenge. While there is potential to reuse digestate as a soil fertilizer or nutrient source for the hydrogenation stage, various obstacles must be addressed, including land constraints and potential negative effects on the bioH_2_-producing microbial consortium from long-term recirculation (Krishnan et al. 2019). To address these challenges, a comprehensive approach is required. This involves developing a reliable gas distribution network with well-equipped filling stations for efficient biohythane delivery. Additionally, establishing enforceable standards and certifications is essential to ensure the safety, quality, and compatibility of biohythane with existing infrastructure. Regarding digestate management, exploring innovative strategies, such as advanced treatment processes and targeted land application techniques, can maximize its value as a resource. Implementing advanced monitoring and control strategies for the recirculation process can help mitigate potential negative impacts on the bioH_2_-producing microbial consortium, ensuring the efficiency and stability of the AD system over the long term.

## Techno-economic and environmental assessments

Conducting a techno-economic assessment is a critical step in the commercialization of biohythane production via the AD process, as it offers valuable insights into the project’s profitability and feasibility (Hans and Kumar 2019). The total project cost comprises the total capital investment and the total annual cost (operating cost) (Jarunglumlert et al. 2018). The total capital investment includes the fixed capital costs of constructing the plant, which encompasses equipment, installation, engineering, construction, and working capital expenses incurred in setting up and operating the plant until revenue is generated (Vo et al. 2018). On the other hand, the total annual cost is the sum of all expenses accrued during the production process, such as raw materials, labor, maintenance, laboratory costs, and so forth (Jarunglumlert et al. 2018). The annual revenue of the biohythane production plant is derived from the sale of biogas and solid waste, as well as waste treatment fees (Jarunglumlert et al. 2018). Once the total costs and annual revenue are estimated, the project’s profitability performance indicators can be determined (Hans and Kumar 2019).

While there is ample research on cost estimates for hydrogen production using various methods such as steam methane reforming, coal and biomass gasification, and electrolysis (Khan et al. 2018), information on the costs of producing bioH_2_ through dark fermentation is limited and requires further investigation. Nevertheless, the production, refining, and bottling of bioH_2_ have similarities with bioCH_4_ (Sasidhar et al. 2022), which could offer insights into cost projections. Therefore, the scalability of biohythane production largely hinges on capital costs, which can potentially be reduced through government incentives like energy efficiency schemes (Mozhiarasi et al. 2023). By providing subsidies and tax benefits, the government can alleviate the financial burden on investors and incentivize them to invest in biohythane production. The initial investment required to establish a two-stage AD process for treating 13.4 tons per hour of biowaste was estimated at 12,687.7 k€ (Ljunggren and Zacchi 2010). Furthermore, research conducted by Micolucci et al. (2018) revealed that processing 27 tons of biowaste per day with a dual-stage thermophilic AD system could yield an annual income of 540,874 €, equivalent to approximately 54.9 € per ton. The payback period for these projects generally ranges from 2 to 6 years, influenced by factors such as the specific composition and properties of the feedstock used in the process (Mozhiarasi et al. 2023).

Another crucial factor that can significantly influence the economic viability of biohythane production is the production scale (Han et al. 2016). As production scale increases, the capital investment cost per unit of production decreases due to economies of scale. This decrease in capital cost can result in a lower overall production cost and a higher profit margin. However, larger-scale production necessitates a greater supply of feedstock, which can raise transportation costs and potentially influence the quality and consistency of the feedstock. Therefore, determining the optimal production scale that balances the advantages of economies of scale with the costs associated with feedstock supply and transportation is essential for the commercial success of biohythane production.

The decision to adopt a two-stage AD process for biohythane production involves balancing the energy output with the added cost of a second digester. Research has shown that the two-stage process yields a positive energy balance compared to single-stage processes for bioH_2_ or bioCH_4_ production, even when factoring in pre-treatment and reactor heating energy requirements (Mozhiarasi et al. 2023). Further, the additional capital investment required for the dual-stage AD in comparison to the single-stage approach is reported to be only 3% (Rajendran et al. 2020), which can be well compensated by the extra energy produced.

In addition to evaluating the economic feasibility of biohythane production, analyzing potential environmental impacts is crucial for its large-scale commercialization. Limited environmental assessments have been conducted on biohythane production via the AD process. In a study by Lembo et al. (2022), the carbon footprints of three different anaerobic digester configurations treating second cheese whey were compared. These configurations included a conventional single-stage anaerobic digester located 50 km from the dairy factory, an on-site single-stage anaerobic digester within the dairy industry premises, and an on-site two-stage system for bioH_2_ and bioCH_4_ recovery. The research findings indicated that the two-stage AD process demonstrated superior energy output, resulting in a 60% reduction in greenhouse gas emissions compared to the off-site single-stage AD. This reduction was primarily attributed to increased bioH_2_ production and enhanced engine performance. Patterson et al. (2013) used an LCA approach to assess the environmental burden associated with the two-stage bioH_2_ and bioCH_4_ production from wheat feedstock, compared to single-stage bioCH_4_ production. The results indicated that the two-stage process had a reduced environmental footprint. They also found environmental advantages in using a bioH_2_-bioCH_4_ blend from the two-stage process as a vehicle fuel over diesel. Sinsuw et al. (2024) conducted a detailed LCA on two-stage biohythane production from livestock wastes at commercial and pilot scales, considering various impact categories. They found that photochemical ozone creation potential had the highest total impact at the commercial scale (33%), while eutrophication potential had the highest impact at the pilot scale (33%). Both biogas production systems had low overall environmental impacts, with the commercial system showing lower impacts than the pilot system. The use of digesters significantly reduced potential environmental impacts related to manure feedstock handling and fertilizer applications. Chen et al. (2020) also utilized an LCA approach to comprehensively assess the energy conversion characteristics and environmental impacts of two-stage biohythane production from microalgae and food waste. The total greenhouse gas emissions from the system were quantified at 173 g CO_2-eq_ MJ^−1^. The main contributors to these emissions were electricity production (41.6%), CO_2_ release during pressurized water processes (27.8%), and energy recovery (19.8%). The carbon fixation process by microalgae significantly decreased net greenhouse gas emissions to 124 g CO_2-eq_ MJ^−1^. Additionally, variations in microalgae growth rate and biohythane yield were identified as key factors influencing greenhouse gas emissions.

Previous studies have also shown that the environmental impacts and advantages of biohythane vary depending on how it is used. For example, research by Liu et al. (2018) employing LCA methodology discovered that biohythane derived from the AD process of corn stalks, when utilized as a vehicle fuel, exhibits a more favorable impact on greenhouse gas emissions compared to alternative options such as combined heat and power (CHP), direct combustion, and compression. Moreover, Bolzonella et al. (2018) discovered that the solid-liquid digestate from the dual-stage biohythane production system has fewer environmental concerns such as acidification and eutrophication due to its lower levels of acid, nitrogen, and phosphorus compared with the conventional single-stage bioCH_4_ production approach.

The techno-economic and environmental assessments discussed above highlight the significant promise of biohythane production through the AD process for sustainable energy and waste management solutions. It is crucial to balance capital investments with operational costs to achieve profitability, with potential reductions possible through government incentives. Moreover, scaling up biohythane production shows clear benefits in terms of economies of scale, despite challenges in feedstock logistics and quality control. Environmental assessments reveal substantial reductions in greenhouse gas emissions and other environmental impacts with the adoption of two-stage AD systems, demonstrating their superiority over conventional methods. These findings underscore the critical role of thorough assessments in guiding the development and deployment of biohythane technologies toward broader commercial viability and environmental sustainability.

## Biohythane integration in waste management systems

The practical application of biohythane production through anaerobic digestion shows great potential for improving waste management practices and providing economic and environmental advantages. However, integrating this technology into existing waste management systems requires careful consideration of several key factors:

### Infrastructure and technological integration

The existing waste management facilities primarily rely on landfill disposal or traditional biogas production, highlighting the necessity for substantial improvements in infrastructure and technology. This involves the implementation of effective pre-treatment units for sorting and treating organic waste, utilizing advanced anaerobic digesters that can produce both bioH_2_ and bioCH_4_, and employing sophisticated gas separation and purification systems to maintain the desired biohythane composition. Addressing these technical requirements is crucial for seamlessly incorporating the new systems into the current infrastructure.

### Economic viability

Assessing the economic feasibility of biohythane production requires a thorough techno-economic analysis. This assessment includes considering initial capital investments, ongoing operational expenses, and market factors affecting hydrogen and methane. Furthermore, potential income from selling hydrogen and methane, as well as government incentives for renewable energy generation and waste disposal, can improve economic feasibility.

### Regulatory and policy framework

Effective regulatory frameworks are essential for the successful implementation of renewable energy solutions. Governments can encourage the production of renewable energy, set quality standards for market acceptance, and implement policies that promote waste diversion to biohythane facilities, thereby reducing the dependence on landfills.

### Environmental impact

Biohythane production supports sustainability objectives by decreasing greenhouse gas emissions and encouraging resource recovery. By redirecting organic waste from landfills to a controlled anaerobic digestion process, methane emissions can be greatly reduced. Biohythane production also allows for the recovery of valuable resources from waste, promoting a circular economy. Furthermore, the concurrent generation of bioH_2_ and bioH_2_ increases the overall energy output from organic waste, offering a more efficient option compared to conventional biogas production.

### Community engagement and awareness

Active community involvement is vital for the successful adaptation of biohythane technology. Public acceptance and participation can be fostered through education and outreach programs that inform communities about the benefits of biohythane and encourage their involvement in waste segregation and collection efforts. Collaboration with local governments, industries, and non-profit organizations can build a supportive network for advancing biohythane production.

By addressing these multifaceted aspects, biohythane is poised to have a significant impact on the waste management sector, providing a sustainable route to a more environmentally friendly future. Moving forward requires conducting pilot projects to demonstrate feasibility, investing in research and development to optimize processes, advocating for supportive policies, and providing capacity building for professionals to effectively manage biohythane production systems. This comprehensive strategy ensures a holistic approach to realizing the full potential of biohythane production.

## Future prospects and possible improvements

The future of biohythane production is poised for significant advancements in efficiency, sustainability, and scalability. Key areas for improvement include:

### Enhanced pre-treatment methods

Effective pre-treatment methods are crucial for converting diverse biomass feedstocks into biohythane. Enzymatic pre-treatment, while environmentally friendly, needs further optimization to address cost and time constraints. Microwave and ultrasound pre-treatment methods show promise for their efficiency, but scalability remains a challenge (Aashabharathi et al. 2024). Future research could focus on developing innovative pre-treatment approaches tailored to different feedstock compositions, optimizing energy efficiency, and reducing processing costs.

### AD process optimization

Optimizing biohythane production involves refining both single-stage and two-stage AD processes. This includes understanding metabolic pathways, scaling-up technical processes, and enhancing reactor performance. Continuous refinement and comprehensive evaluations are essential for commercial-scale biohythane production. Innovations in bioreactor design, especially in integrating advanced monitoring and control systems, can significantly enhance production efficiency. Artificial intelligence (AI) and machine learning (ML) are powerful tools in this endeavor, designed solely on historical data and real-time process information without the need for mathematical models or human intervention. They hold promise, especially in intricate industrial processes where creating mathematical models and obtaining human expertise is challenging (Kazemi et al. 2021). AI-based algorithms like artificial neural networks (ANN) and genetic programming (GP) have proven successful in optimizing process parameters, predicting product yields, and addressing AD issues (Almomani 2020; Gonçalves Neto et al. 2021; Andrade Cruz et al. 2022). Potential future developments could involve the integration of AI and ML for real-time optimization and the development of modular bioreactor systems for decentralized biohythane production.

### Microbial community regulation

Unlocking the full potential of biohythane production requires in-depth knowledge and manipulation of the microbial consortia involved in the process. However, there is a significant lack of comprehensive meta-omics research on AD microbial communities. Multi-omic approaches, such as metagenomics, metatranscriptomics, metaproteomics, and metabolomics, show promise in elucidating microbial functionalities and dynamics, but they necessitate advanced analytical methods and specialized software (Sukphun et al. 2023b). Furthermore, gene manipulation and bio-augmentation strategies are crucial for optimizing AD processes. Cutting-edge genome editing tools like CRISPR/Cas9 and CRISPR/AsCas12a allow for precise genetic modifications (Li et al. 2022; Hao et al. 2023), opening up new possibilities for enhancing AD efficiency and increasing biohydrogen and methane production. Additionally, bio-augmentation, as demonstrated by the development of microbial consortia such as KKU-MC1, has shown potential in enriching microbial communities and enhancing biogas output from challenging biomass sources (Wongfaed et al. 2023). Nevertheless, further research is needed to evaluate the feasibility of bio-augmentation for commercial applications and its integration into the full-scale biohythane production process.

### Utilization of digestate by-products

Leveraging the potential of digestate by-products presents opportunities for resource recovery and circular economy practices. Approaches like composting, nutrient extraction, and biochar production can convert digestate into valuable resources, reducing waste and promoting sustainability (Hung et al. 2017; Vaneeckhaute et al. 2017; Ezemagu et al. 2021). Future efforts could focus on creating integrated biorefinery models to optimize resource extraction from digestate, leading to reduced environmental impact and fostering the sustainable utilization of biomass resources.

### Gas purification, storage, and distribution

Efficient gas purification techniques are essential for maintaining the quality and safety of biohythane. Future advancements could concentrate on creating decentralized purification systems, investigating renewable energy-driven purification technologies, and improving gas storage methods. The utilization of hybrid purification systems, like the membrane-amine hybrid system, can enhance purification effectiveness, decrease energy consumption, and reduce operational expenses. These hybrid systems combine the advantages of various separation processes, addressing the drawbacks of each and enabling more efficient removal of contaminants from the gas stream (Abdulsalam et al. 2019). Furthermore, exploring the on-site integration of hydrogen and methane as a potential method to optimize biohythane quality and usability could be beneficial in the future. This strategy would provide greater control over the gas composition, potentially enhancing the performance and versatility of biohythane in various energy applications. It is also crucial to expand distribution networks to reach remote or underserved areas. Future initiatives should concentrate on expanding infrastructure, integrating smart grid technologies for improved distribution management, and ensuring the reliability and efficiency of biohythane supply chains.

### Techno-economic and lifecycle analyses

While existing evidence suggests the economic viability and environmental benefits of biohythane production, further upscaling studies and comprehensive environmental assessments are necessary. Future research should prioritize refining cost projections and exploring environmental advantages across diverse feedstocks and applications to bolster the advancement and adoption of biohythane production technologies. Recent progress has seen the successful integration of advanced modeling techniques in both techno-economic analysis (Sampat et al. 2022) and lifecycle assessment (Khalaj et al. 2023), enhancing the accuracy and reliability of these evaluations. Future investigations could utilize these sophisticated modeling approaches to analyze the dynamic interactions among technological advancements, market conditions, and policy frameworks. This approach will facilitate informed decision-making and risk management for biohythane projects, ensuring their economic feasibility and environmental sustainability.

### Policy support and market expansion

Supportive policies and incentives are necessary to foster the growth of biohythane as a viable energy source. Measures like investment subsidies, promoting biohythane energy projects, and developing strong supply chains can help expand the market and encourage consumer acceptance. Future policy initiatives could concentrate on creating clear regulatory structures, encouraging research and development in biohythane technologies, and promoting international cooperation to standardize practices and facilitate global trade. These actions would contribute to creating a supportive environment for sustained market growth and competitiveness.

## Conclusion

Biohythane is a promising next-generation biofuel, created by combining bioH_2_ and bioCH_4_ from single-stage or two-stage AD processes. Various microorganisms play key roles in the production of biohythane, each with specific growth and activity requirements. Optimization of parameters such as temperature, pH, retention time, organic loading rate, and nutrient content is crucial for maximizing biohythane yield. Effective recovery is also influenced by substrate type and bioreactor configuration. While the two-stage AD process offers superior energy recovery compared to single-stage methods, challenges like high costs and maintenance impede its widespread commercialization for biohythane production. To achieve success, addressing technical hurdles such as managing microbial consortia, integrating bioH_2_ and bioCH_4_ generation processes, monitoring pH levels, and selecting appropriate pre-treatment methods for complex substrates is essential. Cost-effective biogas purification methods and addressing constraints related to biohythane and by-product utilization are also critical. Furthermore, conducting comprehensive techno-economic and environmental assessments is essential to evaluate the feasibility and sustainability of biohythane production processes. These assessments are pivotal for informing strategic decisions and advancing the viability of biohythane as a renewable energy source.

## Data Availability

Not applicable
